# Upregulated IL-32 Expression And Reduced Gut Short Chain Fatty Acid Caproic Acid in People Living With HIV With Subclinical Atherosclerosis

**DOI:** 10.3389/fimmu.2021.664371

**Published:** 2021-04-15

**Authors:** Mohamed El-Far, Madeleine Durand, Isabelle Turcotte, Etienne Larouche-Anctil, Mohamed Sylla, Sarah Zaidan, Carl Chartrand-Lefebvre, Rémi Bunet, Hardik Ramani, Manel Sadouni, Irina Boldeanu, Annie Chamberland, Sylvie Lesage, Jean-Guy Baril, Benoit Trottier, Réjean Thomas, Emmanuel Gonzalez, Ali Filali-Mouhim, Jean-Philippe Goulet, Jeffrey A. Martinson, Seble Kassaye, Roksana Karim, Jorge R. Kizer, Audrey L. French, Stephen J. Gange, Petronela Ancuta, Jean-Pierre Routy, David B. Hanna, Robert C. Kaplan, Nicolas Chomont, Alan L. Landay, Cécile L. Tremblay

**Affiliations:** ^1^ University of Montreal Hospital Centre (CRCHUM)-Research Centre, Montréal, QC, Canada; ^2^ Département de Microbiologie, Infectiologie et Immunologie, Faculté de Médecine, Université de Montréal, Montréal, QC, Canada; ^3^ Département de Radiologie, Radio-oncologie et Médecine Nucléaire, Faculté de Médecine, Université de Montréal, Montréal, QC, Canada; ^4^ Hôpital Maisonneuve-Rosemont, Montréal, QC, Canada; ^5^ Centre de médecine urbaine du Quartier latin, Montréal, QC, Canada; ^6^ Clinique médicale l’Actuel, Montréal, QC, Canada; ^7^ Department of Human Genetics, Canadian Centre for Computational Genomics, McGill University, Montreal, QC, Canada; ^8^ Microbiome Platform Research, McGill Interdisciplinary Initiative in Infection and Immunity, McGill University, Montreal, QC, Canada; ^9^ Caprion Biosciences Inc., Montréal, QC, Canada; ^10^ Department of Internal Medicine, Rush University Medical Center, Chicago, IL, United States; ^11^ Department of Medicine, Georgetown University, Washington, DC, United States; ^12^ Department of Preventive Medicine, University of Southern California, Los Angeles, CA, United States; ^13^ Cardiology Section, San Francisco Veterans Affairs Health Care System, San Francisco, CA, United States; ^14^ Departments of Medicine, Epidemiology and Biostatistics, University of California San Francisco, San Francisco, CA, United States; ^15^ Division of Infectious Diseases, Stroger Hospital of Cook County, Chicago IL, United States; ^16^ Department of Epidemiology, Bloomberg School of Public Health, Johns Hopkins University, Baltimore, MD, United States; ^17^ Research Institute of McGill University Health Centre, Montréal, QC, Canada; ^18^ Department of Epidemiology and Population Health, Albert Einstein College of Medicine, Bronx, NY, United States; ^19^ Divsion of Public Health Sciences, Fred Hutchinson Cancer Research Center, Seattle, WA, United States

**Keywords:** HIV, CVD (cardiovascular disease), inflammation, atherosclerosis, IL-32, gut microbiome, short-chain fatty acids

## Abstract

Despite the success of antiretroviral therapy (ART), people living with HIV (PLWH) are still at higher risk for cardiovascular diseases (CVDs) that are mediated by chronic inflammation. Identification of novel inflammatory mediators with the inherent potential to be used as CVD biomarkers and also as therapeutic targets is critically needed for better risk stratification and disease management in PLWH. Here, we investigated the expression and potential role of the multi-isoform proinflammatory cytokine IL-32 in subclinical atherosclerosis in PLWH (n=49 with subclinical atherosclerosis and n=30 without) and HIV- controls (n=25 with subclinical atherosclerosis and n=24 without). While expression of all tested IL-32 isoforms (α, β, γ, D, ϵ, and θ) was significantly higher in peripheral blood from PLWH compared to HIV- controls, IL-32D and IL-32θ isoforms were further upregulated in HIV+ individuals with coronary artery atherosclerosis compared to their counterparts without. Upregulation of these two isoforms was associated with increased plasma levels of IL-18 and IL-1β and downregulation of the atheroprotective protein TRAIL, which together composed a unique atherosclerotic inflammatory signature specific for PLWH compared to HIV- controls. Logistic regression analysis demonstrated that modulation of these inflammatory variables was independent of age, smoking, and statin treatment. Furthermore, our *in vitro* functional data linked IL-32 to macrophage activation and production of IL-18 and downregulation of TRAIL, a mechanism previously shown to be associated with impaired cholesterol metabolism and atherosclerosis. Finally, increased expression of IL-32 isoforms in PLWH with subclinical atherosclerosis was associated with altered gut microbiome (increased pathogenic bacteria; *Rothia* and *Eggerthella* species) and lower abundance of the gut metabolite short-chain fatty acid (SCFA) caproic acid, measured in fecal samples from the study participants. Importantly, caproic acid diminished the production of IL-32, IL-18, and IL-1β in human PBMCs in response to bacterial LPS stimulation. In conclusion, our studies identified an HIV-specific atherosclerotic inflammatory signature including specific IL-32 isoforms, which is regulated by the SCFA caproic acid and that may lead to new potential therapies to prevent CVD in ART-treated PLWH.

## Introduction

HIV persistence under ART is associated with non-AIDS comorbidities, including cardiovascular disease (CVD) (1, 2). We and others have previously reported on the increased rate of acute myocardial infarction and coronary heart disease in people living with HIV (PLWH) (3–7), which clearly highlights their vulnerability to CVD and suggests that the underlying mechanisms are likely accentuated in this population (8). Chronic immune activation and inflammation in both untreated and treated PLWH remain central to the pathogenesis of atherosclerosis, the dominant cause of CVD (9–12). In this regard, we have previously reported that expression of the proinflammatory cytokine IL-32 is upregulated in HIV infection and is not normalized with ART, even after long-term of treatment (13). We further showed that IL-32 induces the expression of other inflammatory cytokines such as IL-6, TNF-α and IFN-γ by activated T-cells (13, 14). These observations highlight the key role of IL-32 in sustaining immune activation and chronic inflammation, which are the major etiologic mediators of atherosclerosis (15) and also its potential role as a novel biomarker of CVD. This potential role is further supported by the increased levels of IL-32 in chronic inflammatory diseases linked with CVD such as rheumatoid arthritis (RA), inflammatory bowel disease (IBD) and chronic obstructive pulmonary disease (COPD) (16). However, direct association between IL-32 and CVD remains to be studied. In addition, IL-32 is expressed in different isoforms (α, β, γ, D, ϵ, δ, ζ, η, small/sm, and θ) generated by alternative splicing and showing distinct immune functions varying from proinflammatory to anti-inflammatory and regulatory (13, 17, 18) and it is not yet clear whether all of these isoforms are linked with CVD. To address these questions, we investigated herein the expression of IL-32 isoforms and their potential as CVD biomarkers in PLWH participating in the Canadian HIV and Aging Cohort Study (CHACS) and having coronary artery subclinical atherosclerosis. We also studied the potential mechanisms underlying the persistent upregulation of IL-32 isoforms in peripheral blood from PLWH.

## Materials and Methods

### Definition of Subclinical Atherosclerosis

The current study included HIV+ and HIV^neg^ individuals participating in the Canadian HIV and Aging Cohort Study (CHACS) (mainly men participants) (19). Subclinical atherosclerosis was defined by the presence of atherosclerotic plaque (plaque+) in the coronary arteries, measured using a cardiac computed tomography (CT) scan with injection of contrast media. A 256-slice CT scanner was used (Brilliance iCT, Philips Healthcare, Cleveland, OH, USA). Coronary CT angiography was performed using 370mg/mL iopamidol (Bracco Imaging, Milan, Italy), at a rate of 5 mL/s after bolus tracking. Prospective ECG-gating was used when heart rate was ≤70 bpm otherwise retrospective ECG-gating was used. Gantry rotation time was 270 ms, scan voltage 100 kV for BMI <27 kg/m2 and 120 kV for BMI ≥27 kg/m2, and slice thickness was 0.625 mm. Images were reconstructed using an iterative reconstruction algorithm (Philips iDose4 (level 3), Philips Healthcare). Coronary plaques were identified on the CT angiogram by a board-certified radiologist blinded to the HIV status and clinical data. Plaque volume was assessed using semi-automated software (Aquarius iNtuition 4.4.6, TeraRecon Inc, Foster City, CA, USA), which allows semiautomatic delimitations of plaque borders and, if required, manual adjustment. Excellent inter- and intraobserver agreement for plaque volume has been previously obtained with this technique (intraclass correlation coefficients 0.95 and 0.93, respectively) (20).

### Quantitative PCR for Gene Transcripts

Total RNA was isolated from cryopreserved human peripheral blood mononuclear cells (PBMCs) of ART-treated PLWH and HIV^neg^ individuals using the RNeasy plus mini kit from Qiagen as *per* the manufacturer’s protocol (Catalog #74134). Quantification of IL-32 isoforms (α, β, γ, D, ϵ and θ) was performed using One-step SYBR Green reverse transcription quantitative PCR (RT-qPCR) performed on LightCycler 480II machine (Roche) with QIAGEN QuantiTect (Catalog #204243). Relative expression of IL-32 RNA was normalized to the housekeeping gene β-glucuronidase. Primer sets for the different IL-32 isoforms and β-glucuronidase, conditions for the quantitative PCR and analysis were done as we recently reported (13).

### Soluble Protein Measures in Plasma

Plasma collected from both ART-treated PLWH and HIV^neg^ individuals, with or without subclinical CVD, were treated for at least 1 hour at room temperature with a ratio of 1/5 of disruption buffer (PBS 1X; 0.05% v/v Tween-20, 2.5% v/v Triton X-100, 0.02% v/v thimerosal and 1% v/v trypan blue) to inactivate HIV before cytokine measures. Before running the quantification assays, plasma samples were briefly centrifuged to eliminate cell debris. Total plasmatic IL-32 protein levels were measured with standard ELISA assays using the R&D Systems Human IL-32 DuoSet (Cat #DY3040-05) as *per* the supplier’s protocol. Soluble CD14 (sCD14), LPS-binding protein (LBP), Intestinal Fatty Acid Binding Protein (I-FABP) and LL-37 were quantified using pre-coated ELISA kits from HyCultBiotech (cat #HK320-02, HK315-02, HK406-02 and HK321-02, respectively). The rest of analytes (inflammatory, anti-inflammatory and metabolic markers shown in **Supplemental Table 1**) were quantified with the ultrasensitive Meso Scale Discovery^®^ multiplex kits (a maximum of 10 analytes *per* plate). The assay was performed according to the manufacturer’s protocol. Data analyses were done with the DISCOVERY WORKBENCH 4.0 Software using curve fitting with 4PL.

### Cell Stimulation Assays

Human monocytes were isolated from PBMCs by negative selection using the StemCell EasySep™ Human Monocyte Isolation Kit (cat #19359). Monocytes with purity >95% were stimulated with 500 ng/ml of IL-32 isoforms α, β, or γ (R&D Systems Cat # 3040-IL-050, 6769-IL-025, 4690-IL-025/CF, respectively) in RPMI medium supplemented with 10% FBS. Supernatant of stimulated cells was collected at 48h post-stimulation and cytokines IL-18, IL-β, IL-10, TNF-α and IL-6, were measured by regular ELISA assays using commercial kits from R&D Systems (Cat #DY318-05, DLB50, DY217B-05, DY210, DY206-05, respectively) according to the supplier’s protocol. In another set of experiments, monocytes were stimulated with M-CSF (20 ng/ml) in the presence or absence of IL-32 isoforms and cultured for 3 days. Cultured monocytes were surface-stained with CD14, CD206, CD163, CCR7, and intracellular CD253 (TNF-related Apoptosis-Inducing Ligand (TRAIL)) specific antibodies from BD Biosciences (Cat #555399, 564063, 562643, 557648, 743721, respectively) together with the viability marker Live/Dead stain (Thermofisher Cat #L34957). Stained cells were analyzed on 18-color FACS analyzer (BD). Cell conditioning with short-chain fatty acids and stimulation with LPS were carried out on cryopreserved human PBMCs conditioned for 2 hours with 2mM caproic acid (hexanoic acid, SIGMA, Cat #21529) then stimulated with 200 ng/ml LPS and further incubated for 48 hours.

### Quantification of HIV DNA and Cell-Associated RNA

CD4+ T-cells were enriched by negative selection from cryopreserved PBMCs of ART-treated PLWH men with and without subclinical CVD (n=59) using the EasySep Human CD4+ T-cell Enrichment Kit, according to the supplier’s protocol (StemCell, cat #19052). Purity of isolated CD4+ T-cells was assessed by flow cytometry and was typically >99%. Total DNA and RNA were dually extracted from isolated CD4+ T cells using the AllPrep DNA/RNA Mini Kit, according to the manufacturer’s instructions (Qiagen, cat #80204). The frequency of cells harboring total and integrated HIV DNA was measured from the extracted DNA using our previously described methods (21). Numbers of copies of the CD3 gene were quantified to determine the number of analyzed cells (2 copies per cell). Serial dilutions of extracted HIV DNA from ACH2 cells (1 HIV DNA copy/cell) were used as quantification standards. The cell-associated LTR-gag HIV RNA was quantified by an ultrasensitive semi-nested real-time reverse transcription PCR. Briefly, extracted viral RNA was reverse-transcribed and subjected to 16 cycles of amplification in a Proflex PCR system, followed by a 10-fold dilution and second real-time 45cycles amplification in Rotor-Gene Q. Serial dilutions of LTR-gag *in vitro* transcripts were used as quantification standards. We calculated the number of HIV RNA copies *per* proviral genome for each sample. The primers and probes used for the PCRs are described in **Supplemental Table 2**.

### *In Vitro* Infection

HIV infection of non-activated PBMCs *in vitro* was carried out by Spinoculation (22) using the dual tropic molecular clone HIV-p89.6 (NIH AIDS reagent program Cat #3552) (50 ng HIV-p24/10E6 cells).

### Fecal Collection for Whole Genome Microbiome Sequencing and Targeted Metabolomic Analyses

Fecal samples were collected from n=90 PLWH and HIV^neg^ individuals, with or without subclinical atherosclerosis, participating in the CHACS cohort. Samples were collected at home and were immediately frozen at the participant’s home freezer and then brought to the laboratory in a freezer pack and transferred directly to −80^°^C freezers. For the microbiome studies, n=16 samples were used. Briefly, genomic DNA was quantified using the Quant-iT™ PicoGreen® dsDNA Assay Kit (Life Technologies). Libraries were generated using the NEBNext Ultra II DNA Library Prep Kit for Illumina (New England BioLabs) as *per* the manufacturer’s recommendations. Adapters and PCR primers were purchased from IDT. Size selection of libraries contained the desired insert size has been performed using SparQ beads (Qiagen). Libraries were quantified using the Kapa Illumina GA with Revised Primers-SYBR Fast Universal kit (Kapa Biosystems). Average size fragment was determined using a LabChip GX (PerkinElmer) instrument. The libraries were normalized and pooled and then denatured in 0.05N NaOH and neutralized using HT1 buffer. The pool was loaded at 225pM on Illumina NovaSeq S4 lane using Xp protocol as *per* the manufacturer’s recommendations. Reads were then profiled for microbial species abundances using Metaphlan2 (23) relying on mapping WGS reads to unique clade-specific marker genes from 17,000 reference genomes (including bacterial, archaeal, eukaryotic and viral genomes). Group-wise relative abundance for microbial communities using the hierarchical structure of metaphlan2 taxonomic classifications was analyzed using Heat Tree (24) from Microbiomeanalyst.ca platform (25).

Short-chain fatty acids were measured in fecal samples from the total number of participants (n=90) by LC-MS/MS using a method modified from Han J *et al.*, 2015 (26). Briefly, samples of approximately 25 mg were homogenized manually in 50% aqueous acetonitrile (40 µL *per* mg sample) using a polypropylene pestle and mixed thoroughly for 5 min at 4°C. After centrifugation at 20,000 g, 15 min at 4°C, 30 µL of supernatants, blanks and standards were transferred to glass tubes with 10 µL of a 50% aqueous acetonitrile solution containing 2,2-dimethylbutyric acid as internal standard. Carboxyl groups were derivatized using 3-nitrophenylhydrazine. Derivatized organic acids were separated by reversed-phase chromatography (Nexera X2, Shimadzu) using a C18 column (Poroshell 120 EC-C18, 2.1 x 75 mm, 2.7 µm, Agilent) and detected by ESI-MS/MS in negative-ion mode (QTRAP 6500, SCIEX).

### Statistical Analysis

Data were analyzed using GraphPad Prism 8 (GraphPad software, San Diego, CA). Differences in the same variable between two groups were analyzed with the non-parametric Mann-Whitney test and statistically significant results were considered based on P<0.05 (two-tailed). Analysis of 3 or more groups for the same variable and same treatment was done with ordinary one-way ANOVA and Dunnetts’s multiple comparison test. Data from the same participant before and after treatment were analyzed using Wilcoxon matched pairs test (two-tailed P<0.05 for significance). Correlations between two variables were analyzed with the non-parametric Spearman test (two-tailed P<0.05 for significance). Logistic regressions were performed for each individual variable of interest while including subject's age at visit, smoking and statin medication status as covariates. We used the following formula (*plaque ~ val + `statin use` + `smoking` + `age at visit*`), where *val* corresponds to the z-scores for a given variable, and coronary artery plaque is a binary status (presence/absence) used for the logistic regression. Models were fitted using the maximum likelihood estimation method for generalized linear models, as implemented in the R statistical package (version 4.0.3). Values were centered and scaled to z-score prior to model fitting.

### Ethical Considerations

The study was approved by our Institutional Review Board (IRB) of the “Centre Hospitalier de l’Université de Montréal” Research Center and at all participating sites’ IRBs of the CHACS cohort. All experiments were performed in accordance with the guidelines and regulations approved by the ethic committees from CRCHUM and all IRBs (Ethical approval # CE.11.063). Study participants provided written informed consent for the use of their plasma and cells for research before inclusion in the study and the investigation conformed to the principles outlined in the Declaration of Helsinki.

## Results

### Differential Expression of IL-32 Isoforms in Individuals With Subclinical Atherosclerosis

Expression of IL-32 isoforms was carried out on stored blood collected from n=128 participants from the CHACS cohort including n=79 ART-treated PLWH and n=49 HIV^neg^ men as shown in **Table 1**. Participants were first classified based on the presence or absence of subclinical atherosclerosis in the coronary artery as plaque+ or plaque^neg^, respectively. These participants were then selected based on the criteria of being virologically suppressed by ART (viral load < 40 HIV copies/ml) and having biological samples (blood draw for plasma and PBMCs) from study visits close the date of cardiac imaging. The presence of clinical CVD, ascertained by chart review and self-report, was used as exclusion criteria. Samples from participants with subclinical atherosclerosis were selected from visits with no longer than 12 months prior to the positive cardiac imaging. For individuals who were plaque^neg^, samples from any prior clinical visit (relative to the cardiac imaging date) were used for the analysis (IQR=7 months, Q3-Q1= 7-0 months for HIV+plaque^neg^ and IQR=3 months, Q3-Q1=5-2 months for HIV^neg^plaque^neg^).

**Table 1:** Demographic and clinical parameters of study participants.

Variable	HIV^neg^ plaque- (N=24 males)	HIV^neg^plaque+ (25 males)	*P* value	HIV+plaque- (30 males)	HIV+plaque+ (49 males)	*P* value
Age (Years)	53.04±6.39	55.81±7.47	NS	52.48±6.27	55.55±6.54	0.037
Predicted 10 years Framingham Risk score	10.21±4.36	11.65±4.34	NS	9±4.14	10.69±5.66	NS
Statin Treatment	2 (8.3%)	5 (20%)	NS	4 (13.3%)	19 (38.7%)	0.042
Smoking Never Past Current	15 (62.5%) 7 (29.1%) 2 (8.3%)	12 (48%) 9 (36%) 4 (16%)	0.002	13 (43.3) 12 (40%) 5 (16.6%)	13 (26.5%) 16 (32.6%) 20 (40.8%)	0.01
Body Mass Index Intravenous drug injection Injection No-injection	26.87±3.68 0 (0%) 24 (100%)	27.02±5.68 0 (0%) 25 (100%)	NS N/A	25.8±3.16 1 (3.3%) 29 (96.7%)	24.26±4.84 9 (18.4%) 40 (81.6%)	NS 0.08
Duration of ART (Years)	N/A	N/A		10.95±6.73	14.69±6.81	0.023
Viral load (copies/ml)	N/A	N/A		< 40	< 40*	NS
Nadir CD4 count (Cells/mm^3^)	N/A	N/A		169±164	199±145	NS
CD4 count (Cells/mm^3^)	NA	NA		526±257	594±270	NS
CD4/CD8 ratio	NA	NA		0.85±0.41	0.82±0.44	NS
D-dimer (mg/L)	0.271±0.160	0.371±0.112	NS	0.416±0.349	0.340±0.166	NS
hsCRP (mg/L)	NA	NA		5.86±5.16	6.58±11.76	NS
LDL–C (mmol/L)	3.37±0.8	3.07±1.12	NS	3.03±0.67	2.65±0.87	0.031
HDL–C (mmol/L)	1.39±0.458	1.29±0.27	NS	1.22±0.34	1.23±0.37	NS

NA, Non-available. *Two individuals had viral load of 92 and 76 copies/ml. Numbers are shown in Mean ± SD. N/A: non-applicable.

NS, Non-Significant.

**Figure 1:**
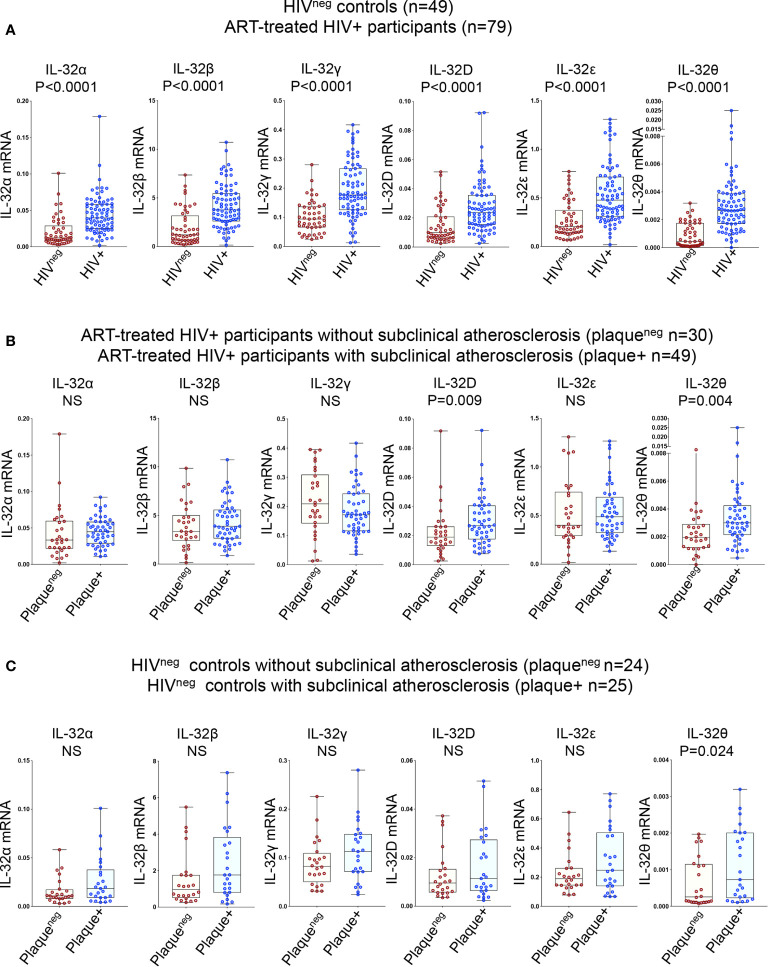
Relative expression of IL-32 isoforms in HIV+ and HIV^neg^ individuals (with or without subclinical atherosclerosis) shown in Box-whiskers plot (min-max). **(A)** RT-qPCR data for IL-32 isoforms (α, β, γ, D, ϵ and θ) amplified from total PBMCs of HIV^neg^ (n=49) compared to HIV+ individuals (n=79). **(B)** HIV+ individuals without or with subclinical atherosclerosis (n=30 plaque^neg^ and n=49 plaque+, respectively). **(C)** HIV^neg^ individuals without or with subclinical atherosclerosis (n=24 plaque^neg^ and n=25 plaque+, respectively). IL-32 RNA levels were normalized to the housekeeping gene β-glucuronidase. *P* values are calculated with the non-parametric Mann-Whitney test. NS, non-significant.

Given the multitude of IL-32 isoforms (27) and the lack of specific antibodies to distinguish between these isoforms in plasma, we quantified the expression of individual IL-32 isoforms at the transcriptional level. Six IL-32 isoforms (α, β, γ, D, ϵ, and θ) were quantified in total PBMCs isolated from the study participants. Consistent with our previous data on independent samples (13), we confirmed that expression of all IL-32 isoforms was significantly higher in HIV+ individuals compared to HIV^neg^ controls (**Figure 1A**). IL-32 expression was then separated based on the presence or absence of subclinical atherosclerosis in both groups. As shown in **Figure 1B**, expression of IL-32D and IL-32θ isoforms was significantly higher in HIV+ individuals with subclinical atherosclerosis (herein referred to as HIV+plaque+) compared to their HIV+plaque^neg^ counterparts (p=0.009 and p=0.004, respectively). However, in the HIV^neg^ controls, only IL-32θ was significantly upregulated in the plaque+ compared to the plaque^neg^ populations (p=0.024) (**Figure 1C** **)**. These results suggest that expression of IL-32 isoforms may represent a signature relevant to subclinical atherosclerosis in both HIV+ and HIV^neg^ individuals.

### HIV-Specific Subclinical Atherosclerotic Plasmatic Signature Correlating With IL-32 Expression

**Figure 2:**
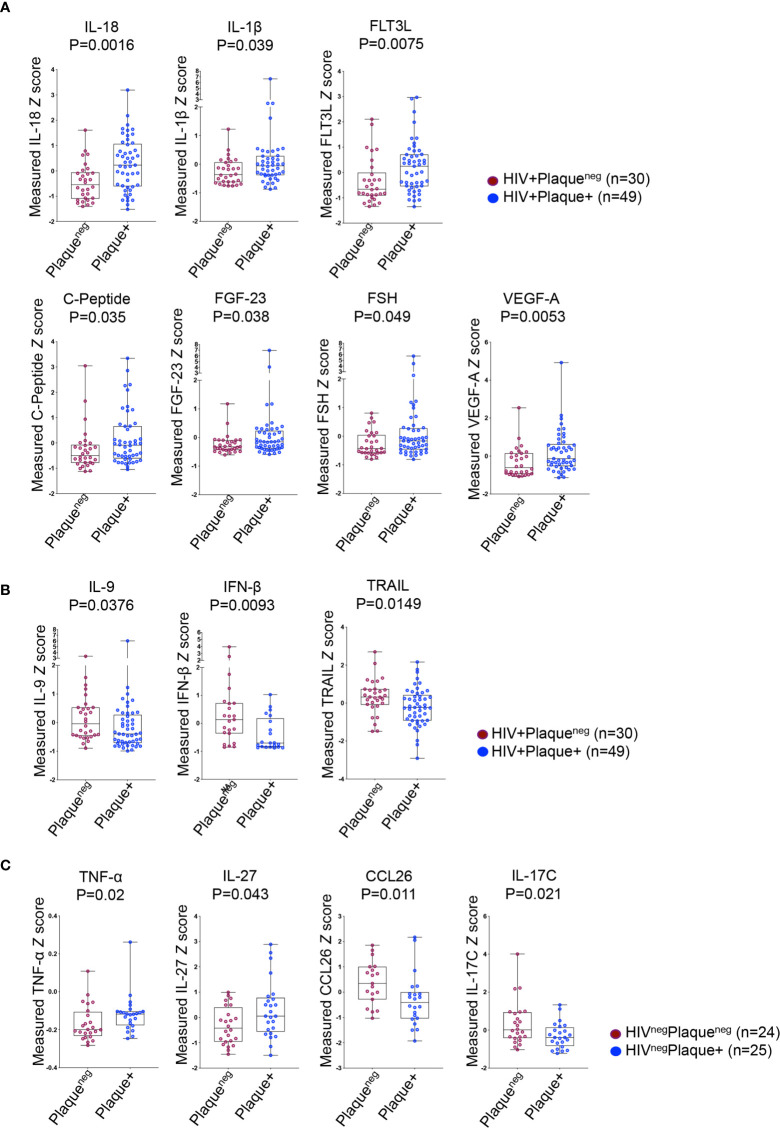
Differentially expressed plasma analytes in HIV+ and HIV^neg^ participants with or without subclinical atherosclerosis shown in Box-whiskers plot (min-max) of analytes *Z* scores. **(A)** Upregulated analytes in HIV+ individuals without (n=30 plaque^neg^) compared to HIV+ individuals with subclinical atherosclerosis (n=49 plaque+). **(B)** Downregulated analytes in HIV+ individuals as in A. **(C)** Upregulated (left panels) and downregulated analytes (right panels) in HIV^neg^ without (n=24 plaque^neg^) compared to HIV^neg^ individuals with subclinical atherosclerosis (n=25 plaque+). All analytes were measured with Meso Scale Discovery technology from plasma. *P* values are calculated with the non-parametric Mann-Whitney test.

We further investigated the association between IL-32 and other inflammatory factors known to be linked with higher CVD risk such as IL-6, IFNγ and TNF-α (28). We quantified a panel of 88 inflammatory, anti-inflammatory and metabolic analytes measured from the same individuals. Analytes with a performance of less than 50% successful detection were excluded from the analysis (**Supplemental Table 1** **)**. For better comparability between analytes, we generated the standardized *Z*-score values for each candidate. Among all tested candidates, ten analytes were differentially expressed between HIV+ individuals with or without subclinical atherosclerosis, whereas only four analytes in the HIV^neg^ group were differentially expressed. In the HIV+ group, we observed a specific signature characterized by higher plasma levels of IL-18, IL-1β, FLT3L, C-peptide, FGF-23, FSH and VEGF-A in individuals with compared to those without subclinical atherosclerosis (p=0.0016, 0.039, 0.0075, 0.035, 0.038, 0.049 and 0.0053, respectively, **Figure 2A**) combined with lower levels of IL-9, IFNβ and the TNF-related apoptosis inducing ligand (TRAIL) (p=0.0376, 0.0093 and 0.0149, respectively, **Figure 2B**). However, the subclinical atherosclerosis-associated plasmatic signature in the HIV^neg^ population was different and marked with the upregulation of TNF-α and IL-27 and downregulation of CCL26 and IL-17C (p=0.02, 0.043, 0.011 and 0.021, respectively) (**Figure 2C**).

Differentially expressed analytes between individuals with or without subclinical atherosclerosis (including IL-32 isoforms) were then verified against the presence of outliers using parameter estimation by regression (29) and considering outliers with a *z*-score >+3 or <-3 of standard deviation of the dataset. While we did not observe systemic outlier samples, statistical analysis of three analytes (IL-1β, FGF-23, and FSH) became marginally significant, only in the HIV+ group (p=0.053, 0.068 and 0.085, **Table 2**). Furthermore, we carried out independent logistic regressions on each of these variables together with the different measured IL-32 isoforms while correcting for subject's age at visit, smoking and statin treatment status. By using this analysis in the HIV+ individuals, we observed that expression of IL-32D and IL-32θ, IL-18, VEGF-A and IFNβ remained significantly associated with coronary artery atherosclerosis independently of age, smoking or statin treatment (p=0.031, p=0.042, p=0.001, p=0.029 and p=0.046, respectively), whereas TRAIL, IL-1β and FLT3L showed borderline significance (p=0.052, p=0.055, and 0.059, respectively). In the HIV^neg^ group, IL-27, IL-17C and CCL26 remained significantly associated with subclinical atherosclerosis following the regression analysis (p=0.041, p=0.012 and p=0.016, respectively), whereas IL-32θ and TNF-α were no longer significant (**Table 2**). Together, these results suggest that the signature of IL-32 isoforms together with IL-18, VEGF-A, IFNβ, TRAIL and IL-1β correlate with subclinical atherosclerosis in HIV infection.

**Table 2:** Verifications of outliers and adjustment for age, smoking and statin treatment for the differentially expressed analytes between individuals with/without coronary artery atherosclerosis.

Analyte	*P* value/Outlier- verified *P* value	Odds ratio/SD increase	95%CI	Adjusted *P* value
**HIV+ (n=30 Plque^neg^/n=49 plaque+)** *Upregulated analytes*				
IL-32D*	0.009/0.018	2.002	1,118-3,999	0.031
IL-32θ*	0.004/0.008	3.666	1,203-14,184	0.042
IL-18*	0.0016/0.002	2.619	1,493-5,090	0.001
IL-1β	0.039/0.053	3.333	1,190-12,98	0.055
FLT3L	0.0075/ND	1.726	1,015-3,215	0.059
C-Peptide	0.035/0.047	1.502	0,858-2,904	0.183
FGF-23	0.038/0.068	2.674	1,011-13,58	0.171
FSH	0.049/0.085	1.962	0,955-5,693	0.151
VEGF-A*	0.0053/0.007	2.134	1,144-4,536	0.029
*Downregultated analytes*				
IL-9	0.0376/0.0386	0.701	0,365-1,162	0.212
IFNβ*	0.0093/0.0153	0.485	0,209-0,884	0.046
TRAIL	0.0149/ND	0.582	0,323-0,977	0.052
**HIV^neg^ (n=24 Plque^neg^/n=25 plaque+)** *Upregultated analytes*				
TNF-α	0.02/0.033	638.05	1.570-2451888	0.086
IL-27* IL-32θ	0.043/ND 0.024/ND	2.205 1.742	1.098-5.165 0.957-3.392	0.041 0.079
*Downregultated analytes*				
CCL-26*	0.011/ND	0.359	0.138-0.768	0.016
IL-17C*	0.021/0.035	0.300	0.103-0.697	0.012

SD, Standard deviation for each analyte; ND, Non-detected. * Analytes remaining significant after adjustment.

### IL-32 Activates Monocytes and Induces an M1 Inflammatory Macrophage Phenotype

**Figure 3:**
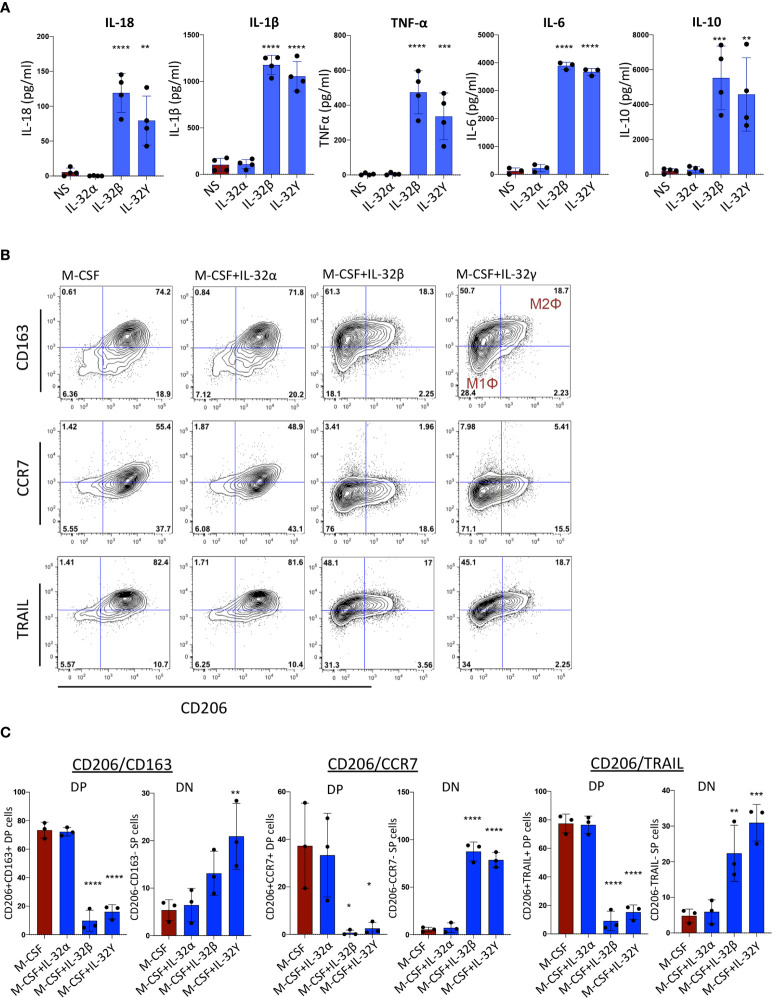
Monocyte activation and differentiation with IL-32 isoforms. **(A)** Bar graphs showing mean ± standard deviation for cytokine production of IL-18, IL-1β, TNF-α, IL-6 and IL-10 measured by ELISA from supernatant of monocytes stimulated with IL-32α, IL-32β or IL-32γ for 48h (n=4). **(B)** Flow cytometry analysis showing phenotype of monocytes stimulated with M-CSF in the presence or absence of IL-32α, IL-32β or IL-32γ for 3 days (n=3); co-expression of CD206 and CD163 (upper panels), co-expression of CD206 and CCR7 (middle panels) and CD206 and TRAIL (lower panels). Live cells were gated based on Live/dead discriminator and CD14 expression. **(C)** Graphs showing mean ± standard deviation for the double positive (DP) and double negative (DN) populations for CD206 and CD163 (left panels), CD206 and CCR7 (middle panels) and CD206 and TRAIL (right panels). Data analyzed with ordinary one-way ANOVA and Dunnetts’s multiple comparison (*P≤0.05, **P≤0.01, ***P≤0.001, **** P≤0.0001). NS, Non-stimulated conditions.

Among the identified plasmatic proteins associated with subclinical atherosclerosis, we were particularly interested in the upregulation of IL-18 and the corresponding downregulation of TRAIL. In a recent study, IL-18 was shown to repress TRAIL expression in monocytes/macrophages leading to a blockade in reverse cholesterol transport and exacerbation of inflammation and atherosclerosis (30). In addition, we observed positive correlations between plasmatic IL-18 levels and RNA transcripts of the different IL-32 isoforms (**Supplemental Figure 1**). Next, we evaluated the impact of IL-32 isoforms on induction of IL-18 in monocytes. Since IL-32D and IL-32θ are not yet commercially available, we used IL-32α, β and γ proteins (R&D Systems) in the functional assays. Of note, IL-32D shares 95% of protein sequence homology with the dominant IL-32β isoform and similar pro-inflammatory functions are expected as we previously reported (13). As shown in **Figure 3A**, a significant increase in IL-18 as well as IL-1β, TNF-α, IL-6 and IL-10 was observed when human monocytes were stimulated *in vitro* with IL-32 isoforms β and γ, whereas IL-32α did not activate monocytes. Interestingly, monocytes stimulated with IL-32γ and to a lower extent with IL-32β, acquired an inflammatory macrophage M1Φ phenotype (CD206^neg^CD163^neg^) compared with cells stimulated with M-CSF alone or in combination with IL-32α, which were distinguished by an anti-inflammatory M2Φ phenotype (CD206+CD163+). In addition, monocytes stimulated with IL-32β and IL-32γ significantly downregulated both TRAIL and CCR7 (**Figures 3B, C**). Together, these results link IL-18-mediated downregulation of TRAIL (an atherosclerosis-promoting mechanism (30)) with upregulated IL-32 expression and suggest an upstream regulatory role for IL-32 in this process.

### Potential Role of HIV-1 Reservoir and Bacterial Translocation in Upregulation of IL-32 Expression in PLWH

**Figure 4:**
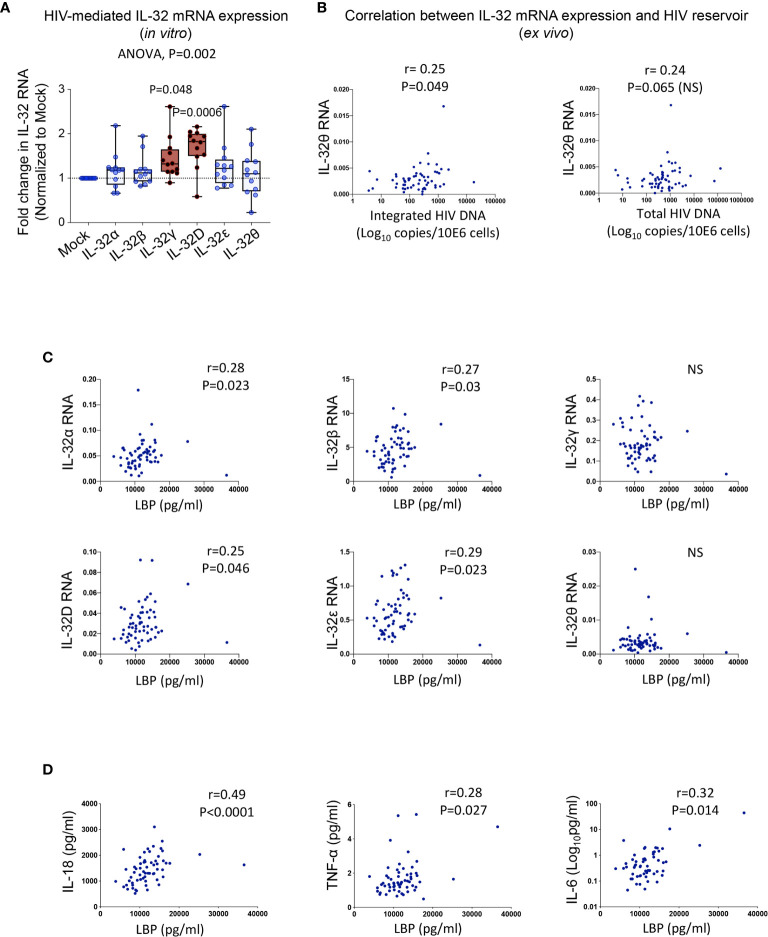
Potential inducers of IL-32 in HIV infection. **(A)** Box-whiskers plot (min-max) for expression of IL-32 isoforms α, β, γ, D, ϵ and θ (measured by RT-qPCR and normalized to the housekeeping gene β-glucuronidase) in non-stimulated PBMCs isolated from healthy donors (n=12) and exposed to the dual tropic (X4/R5) HIV clone p89.6 for 6h. **(B)** Correlation between IL-32θ isoform and levels of integrated HIV DNA (left panel) and total HIV DNA (right panel) measured in primary CD4+ T-cells isolated from ART-treated HIV+ individuals with or without subclinical atherosclerosis (n=59) and expressed as Log_10_ copies *per* million cells. **(C)** Correlations between plasmatic LBP levels and cell-associated IL-32 isoform RNA from n=61 HIV+ individuals. **(D)** Correlations between LBP and IL-18 (Left panel), TNF-α (middle panel) and IL-6 (right panel) from the same individuals as in **(C)**. Data analyzed with ordinary one-way ANOVA and Dunnetts’s multiple comparison in **(A)** and non-parametric Spearman correlations in **(B–D)**. NS, non-significant.

To gain insight into the mechanism(s) underlying the upregulated expression of IL-32 isoforms in PLWH, we first studied the effect of HIV infection in primary cells. Human PBMCs from non-infected individuals were exposed to HIV without prior cell activation to avoid modulation of IL-32 expression. Although HIV induced the expression of all IL-32 isoforms, only IL-32D and IL-32γ reached statistical significance (**Figure 4A**). Given this HIV-mediated IL-32 expression *in vitro*, we hypothesized a link between the HIV reservoir and increased IL-32 expression in ART-treated PLWH. To test this hypothesis, we quantified integrated and total HIV DNA levels as well as cell-associated HIV RNA in primary CD4+ T-cells from a subset of the same study participants in whom IL-32 and other CVD biomarkers were measured (n=59). IL-32θ, one of the two subclinical atherosclerosis-associated IL-32 isoforms, showed a statistically significant correlation with integrated HIV DNA (r=0.25, p=0.049), a marginally significant correlation with total HIV DNA (r=0.24, p=0.065), but no correlation with cell-associated HIV RNA (**Figure 4B**). The correlation between the size of HIV reservoir and IL-32θ but not IL-32D isoform suggests that other persistent stimulator(s) might be involved in the upregulation of IL-32 isoforms under subclinical atherosclerotic conditions. In this regard, gut dysbiosis and bacterial translocation are known to be a major source of persistent peripheral inflammation with bacterial LPS being a potential inducer of IL-32 expression (31–33). Interestingly, our data showed that plasma LPS-binding protein, LBP (a surrogate marker for bacterial LPS presence in circulation), from PLWH correlated with RNA expression of IL-32D together with IL-32α, β, and ϵ (**Figure 4C**). LBP also positively correlated with the inflammatory cytokines IL-18, TNF-α and IL-6 from the same individuals (**Figure 4D**). These observations suggest that upregulation of the atherosclerosis-associated IL-32 isoforms in ART-treated PLWH might be driven, at least in part, by HIV DNA and bacterial translocation.

### Association Between Gut Microbiome and IL-32 in PLWH With Subclinical Atherosclerosis

**Figure 5:**
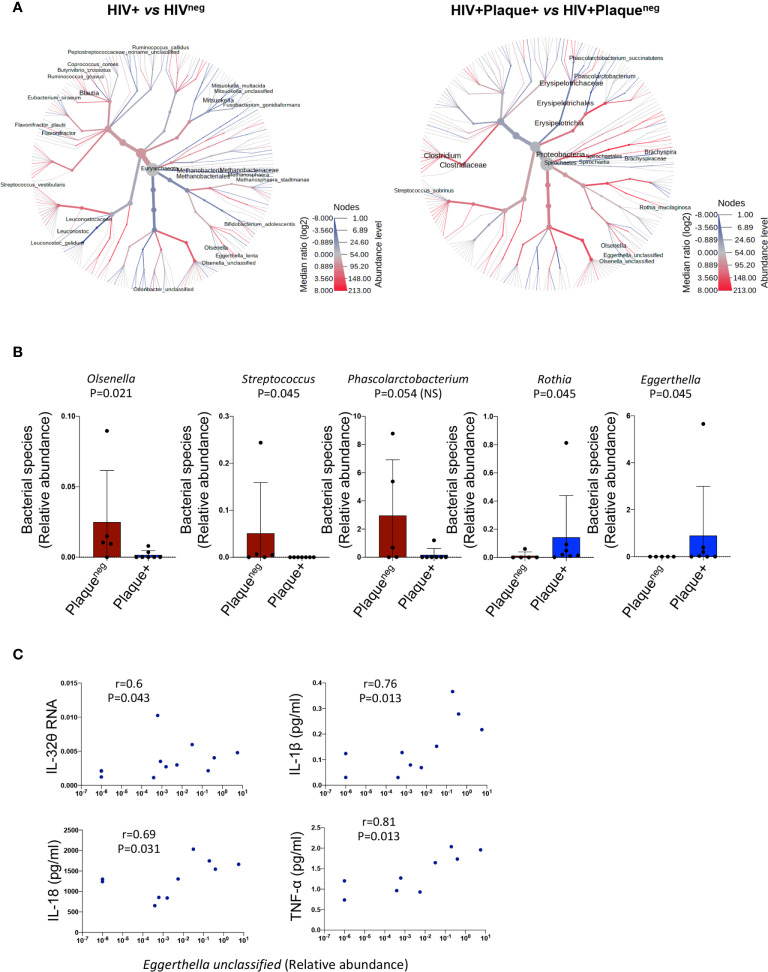
Difference in microbiome composition in HIV+ participants with or without subclinical atherosclerosis. **(A)** Heat tree analysis showing group-wise relative abundance for microbial communities using the hierarchical structure of metaphlan2 taxonomic classifications in HIV+ (n=12) compared to HIV^neg^ participants (n=4) (left panel) and HIV+ with (n=7) compared to HIV+ participants without subclinical atherosclerosis (n=5) (right panel). **(B)** Bar graphs showing mean ± standard deviation for the significantly decreased bacterial species (left panels) and the significantly abundant species (right panels) in HIV+ participants with (n=7) compared to their counterparts without subclinical atherosclerosis (n=5). **(C)** Correlations between the bacterial species *Eggerthella* and IL-32θ, IL-1β, IL-18 and TNF-α in the HIV+ group (with and without subclinical atherosclerosis). Non-parametric Mann-Whitney test was used to compare metaphlan2 inferred genera between Plaque^neg^ and Plaque+ groups in **(B)** and non-parametric Spearman was used to test correlations in **(C)**.

Given the link between LBP, and hence LPS, and the subclinical atherosclerosis-associated IL-32 isoforms, we aimed to determine whether gut microbiome composition is distinct in PLWH under subclinical atherosclerosis. Sixteen individuals from the CHACS cohort were recruited for fecal collection and microbiome metagenomics (randomly selected from HIV+plaque+ [n=7], HIV+plaque^neg^ [n=5] and HIV^neg^ [n=4]). Using a heat tree map (**Figure 5A**, left panel and **Supplemental Table 3i**) we show the community structure of bacterial and archaea species with modulated abundance in HIV+ compared to HIV^neg^ individuals. Among these candidates, 12 species were significantly decreased (consistent with the reported decreased α diversity in HIV infection (34)) and only 3 species were enriched. By limiting the analysis to HIV+ individuals with or without subclinical atherosclerosis (**Figure 5A** right panel and **Supplemental Table 3ii**), two species were significantly decreased (*Olsenella unclassified and Streptococcus sobrinus*), whereas 2 other species were enriched (*Rothia mucilaginosa* and *Eggerthella unclassified*) **(** **Figure 5B** **)**. Interestingly, abundance of the *Eggerthella unclassified* species positively correlated with IL-32θ, IL-1β, IL-18, and TNF-α levels (**Figure 5C**, full dataset of microbiome sequencing is available through the NCBI Sequence Read Archive (SRA) under the accession number PRJNA713013)). Together, these results suggest that the *Eggerthella* species might be involved in the persistent upregulation of IL-32 and atherogenesis in PLWH.

### Lower Levels of Gut Short-Chain Fatty Acids Are Linked With IL-32 Expression and Subclinical Atherosclerosis

**Figure 6:**
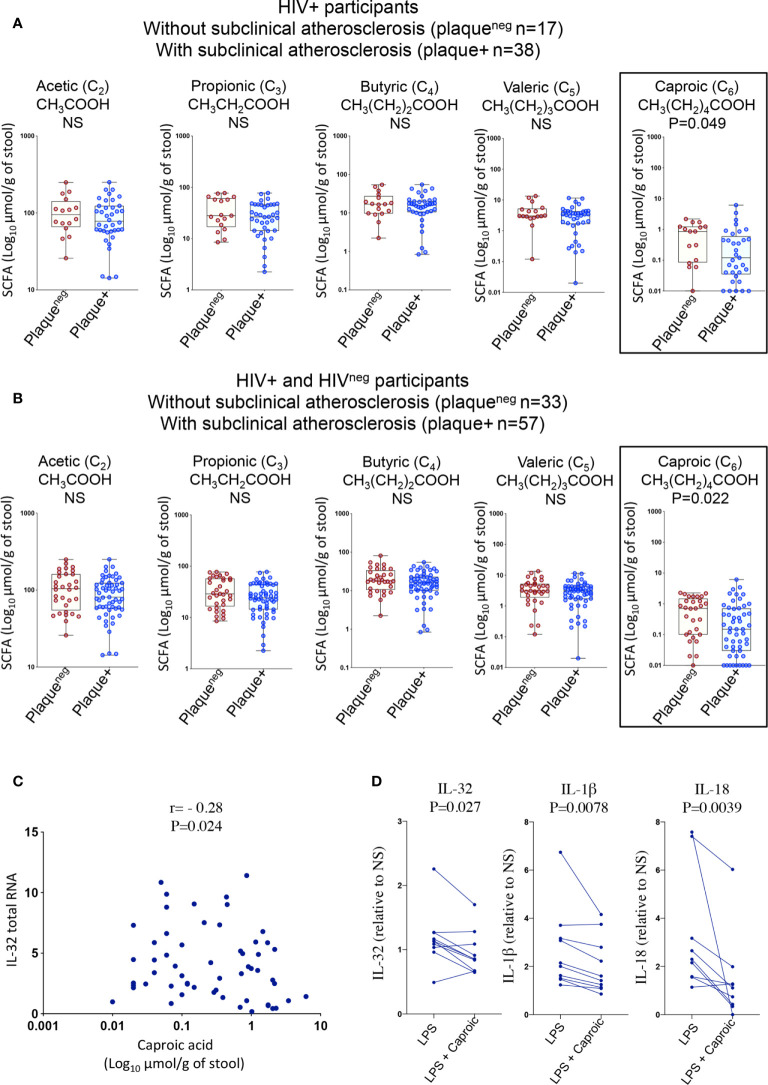
Gut levels of short-chain fatty acids in HIV+ and HIV^neg^ participants with or without subclinical atherosclerosis shown in Box-whiskers plot (min-max). **(A)** Levels of the individual short chain fatty acids measured in fecal samples collected from HIV+ individuals without (n=17 plaque^neg^) compared to HIV+ participants with subclinical atherosclerosis (n=38 plaque+). **(B)** Levels of the individual short chain fatty acids measured in fecal samples collected from both HIV+ and HIV^neg^ without (n=33 plaque^neg^) compared to individuals with subclinical atherosclerosis (n=57 plaque+). All values are expressed in Log_10_ μmol/g of fecal sample. **(C)** Correlation between levels of caproic acid in fecal samples and IL-32 total RNA in PBMCs (measured by RT-qPCR and normalized to the housekeeping gene β-glucuronidase) from HIV+ and HIV^neg^ participants (n=63). **(D)** Impact of caproic acid pre-treatment (2mM for 2 hours) on production of inflammatory cytokines IL-32, IL-1β and IL-18 in PBMCs stimulated with LPS (n=10). Cytokines measured by ELISA in supernatants of stimulated cells following 48 hours of stimulation (IL-18 and IL-1β) or in total cell lysate (for cell-associated IL-32 protein). Data are expressed as a fold change in cytokine expression in LPS-stimulated PBMCs, conditioned or not with caproic acid, relative to non-stimulated cells (NS). Data analyzed with the non-parametric Mann-Whitney in *A, B* Spearman correlation in *C* and Wilcoxon matched-pairs rank test in *D*. NS, non-significant.

Given the significant changes in gut microbiota composition and the association with inflammatory mediators, we thought to test whether these changes were further associated with the functional metabolome. We therefore investigated gut levels of short-chain fatty acids (SCFAs); the main products of saccharolytic fermentation of dietary fibers by gut microbiota, which play multiple key roles as both energy source and modulators of mucosal immunity and inflammation (35, 36). We quantified fatty acids with 2-6 carbon atoms corresponding to short-chain fatty acids (acetic (C_2_), propionic (C_3_), butyric (C_4_), valeric (C_5_) and caproic (C_6_) (37)) in fecal samples from HIV+ and HIV^neg^ individuals (n=90, **Supplemental Table 4**) among whom, n=63 participated in the IL-32 and biomarker studies described in **Figures 1** and **2**. As shown in **Figure 6A**, HIV+plaque+ individuals showed a tendency for a lower abundance of gut SCFA levels when compared to their counterparts of HIV+plaque^neg^ individuals. Similar results were also observed with HIV^neg^plaque+ compared to HIV^neg^plaque^neg^ individuals **(** **Supplemental Figure 2A**). However, these differences did not reach statistical significance except for the caproic acid (hexanoic acid), which was less abundant in the HIV+plaque+ group (p=0.049). This difference became more significant when the study participants (both HIV+ and HIV^neg^) were grouped together and stratified based on their subclinical atherosclerosis conditions, regardless of the HIV status, where the presence of coronary artery atherosclerosis was associated with lower abundance of caproic acid (p=0.022, **Figure 6B**). Interestingly, caproic acid levels in the fecal samples negatively correlated with IL-32 total RNA measured from the n=63 matched donors (**Figure 6C**, r= -0.28, p=0.024) as well as with the individual IL-32 isoforms (**Supplemental Figure 2B**). These data suggest that caproic acid might play a protective role against IL-32 upregulation. To test this hypothesis, we induced IL-32 expression with LPS in total PBMC, isolated from control individuals, in the presence or absence of caproic acid. As shown in **Figure 6D**, pre-treatment of PBMC with caproic acid diminished the expression of cell-associated IL-32 protein (p=0.027) as well as the production of IL-1β (p=0.0078) and IL-18 (p=0.0039). Together, our data point to the SCFA caproic acid as an important modulator of inflammation; likely protective against the development of the inflammation-driven atherosclerosis in ART-treated PLWH.

## Discussion

In the current study, we identified the human cytokine IL-32 as a key inflammatory player associated with subclinical atherosclerosis in PLWH. The upregulation of specific IL-32 isoforms together with IL-18 and IL-1β and down-regulation of TRAIL in these individuals in the absence of acute cardiovascular events suggests that these inflammatory mediators are involved in the pathogenesis of the silent process of atherogenesis. Upregulation of IL-32 is tightly regulated by both positive and negative feedback mechanisms aiming to balance anti-viral and anti-bacterial responses while avoiding deleterious effects of its persistent expression (38, 39). This tight regulation seems to be compromised in HIV infection since IL-32 is not normalized with ART and consequently shows persistent upregulation (13, 14). However, not all IL-32 isoforms are expected to contribute to the chronic inflammation. For instance, we and other have shown that while IL-32β (the dominant isoform in human PBMCs (13)) and IL-32γ are strong inducers of other proinflammatory cytokines including IL-6, IFNγ and TNF-α, IL-32α may exert anti-inflammatory functions by inducing IL-10 in activated T-cells (13, 14, 40). This was in line with earlier reports showing the anti-atherosclerotic potential of IL-32α as it inhibits endothelial inflammation and vascular smooth muscle cell activation by upregulating Timp3 and Reck through suppressing microRNA-205 (41). Given this multitude of IL-32 isoforms that are associated with different functions together with the lack of specific antibodies to distinguish these isoforms at the protein level, we studied their expression at the transcriptional level. Importantly, we have previously shown that IL-32 mRNA levels positively and significantly correlate with cell-associated IL-32 protein detected by a set of antibodies that recognize the total pool of IL-32 proteins (13). Indeed, in our current study, we observed that all tested IL-32 isoforms were higher in HIV+ compared to HIV^neg^ individuals. However, by limiting the comparison between HIV+ individuals with and without subclinical atherosclerosis, only two isoforms (IL-32D and θ) were differentially expressed. Of note, IL-32D shares 95% of protein sequence homology with the dominant IL-32β isoform and similar pro-inflammatory functions are then anticipated. However, for IL-32θ, early studies suggested an anti-inflammatory role as it reduces IL-1β production by attenuating phosphorylation of PU.1 through its interaction with PKCδ (42). Yet, IL-32θ is expressed at a much lower rate compared to IL-32D (10 times less (13)) and the functional relevance of these lower rates remains to be determined. Of note, the expression and association of IL-32 isoforms with subclinical CVD in the current study was carried out in blood collected from HIV+ men in the CHACS cohort, which predominantly recruits men participants. However, it is important to mention that we also observed differential expression and association between specific IL-32 isoforms and subclinical atherosclerosis in HIV+ women participating in the Women Interagency HIV Study, (WIHS Cohort). Interestingly, IL-32 expression of all the different isoforms was significantly higher in HIV+ men compared to HIV+ women (data not shown). Whether this higher expression in men compared to women is sex-related or linked to ethnicity and whether it is related to increased risk for CVD is not yet clear and studies are ongoing to address these questions.

To better understand the functional consequences of IL-32 upregulation in subclinical atherosclerosis among HIV+ individuals, we screened a large panel of cytokines and inflammatory biomarkers known to induce or to be induced by IL-32 such as IL-6, TNF-α, sCD14 and IL-8 (14, 43). This screening allowed us to identify a plasmatic signature associated with subclinical atherosclerosis specific to PLWH that included the upregulation of IL-18 and IL-1β and downregulation of TRAIL, three important proteins with known functions in atherogenesis (30, 44, 45). The differential expression of these factors together with IL-32 isoforms D and θ in HIV+ individuals with and without subclinical atherosclerosis was independent of age, smoking and statin treatment. However, we acknowledge the limitation of the relatively modest numbers of HIV+ individuals *per* tested group (n=49 with subclinical atherosclerosis and n=30 without) that restricted further statistical adjustment for other potential confounders. Future studies will include higher numbers of participants, including women, to confirm our observations.

IL-32 expression showed a marked correlation with IL-18 and IL-1β, two effector cytokines of the NLRP3 inflammasome that is believed to be a novel mediator of CVD (46, 47). The link between IL-32 and IL-18 was previously reported by earlier studies showing that IL-32 upregulation in human PBMCs in response to LPS or *Mycobacterium tuberculosis* is mediated by activation of Caspase-1 and IL-18 components of the inflammasome (33). In the current study we further found that IL-32 induces IL-18 expression in activated monocytes, which suggests a positive feedback loop between these two inflammatory cytokines. In fact, this link is of particular interest given the key role of IL-18 in modulating cholesterol metabolism in monocytes/macrophages where it induces the down-regulation of TRAIL by reducing NF-κB binding activity on the TRAIL promoter. Down-regulation of TRAIL in monocytes/macrophages turns these cells into lipid-laden reservoirs and enhances their inflammatory capacity in response to LPS stimulation and their atherogenic potential (30). In the current report, we established the link between IL-32 and TRAIL by *in vitro* studies showing decreased TRAIL expression in monocytes stimulated with IL-32 isoforms and the upregulation of IL-18 by these cells. Such a mechanism may largely contribute to the atherogenic process since TRAIL^neg^ macrophages produce higher levels of TNF-α, IL-1β, and IL-6 mRNA and also the potent monocytes attractant CCL2 (30). The effect of IL-32 on monocytes/macrophages was also associated with two important phenotypic changes; the down-regulation of the mannose receptor CD206 and CD163 indicative of the acquisition of an M1Φ-like phenotype (inflammatory profile) and the down-regulation of CCR7. Interestingly, CCR7 down-regulation may impact the potential egress of activated monocytes/macrophages from the site of atherosclerotic lesion, which would then enhance local inflammation and foster atherogenesis (48, 49). Indeed, the activation of CCR7-dependent immigration of monocytes from the lesion site was previously suggested to be one of the mechanisms by which statin treatment can decrease atherosclerosis (49).

Since our data showed a highly significant potential role for IL-32 in atherogenesis, it was important to identify the underlying mechanisms by which IL-32 upregulation is sustained in ART-treated PLWH. While association between persistent IL-32 mRNA expression and the size of HIV DNA reservoir was limited to IL-32θ (one of the two subclinical atherosclerosis-associated isoforms), significant correlations between LBP and the majority of IL-32 isoforms suggested a potential role for gut microbiota and/or microbiota metabolites on IL-32 persistent expression. Consistent with earlier studies (50), we observed a decrease in the diversity of gut microbiota in PLWH compared to HIV^neg^ individuals. However, when restricting the analysis to HIV+ participants, only a limited number of bacterial species were associated with subclinical atherosclerosis. Two species showed significant lower abundance; *Streptococcus sobrinus* and *Olsenella unclassified*, whereas two other bacterial species (*Rothia mucilaginosa* and an *Eggerthella unclassified* species) were significantly abundant in subclinical atherosclerosis conditions. Interestingly, correlations with the inflammatory factors were only observed with the *Eggerthella unclassified* species that positively correlated with TNF-α, IL-18, IL-1β and IL-32θ. This is in line with the pathogenic potential of these bacteria as members of the *Eggerthella* genus are linked with chronic inflammatory diseases such as rheumatoid arthritis and Crohn’s disease (51, 52). To better understand the functional consequences of this gut dysbiosis, it was important to consider the associated metabolic pathways as we and others suggested earlier (32). Among the potential mechanisms by which the compromised gut microbiota diversity may fuel and sustain inflammation is being through the biased metabolism of non-digestible fiber-rich diets to generate short-chain fatty acids (fatty acids with up to 6 carbon atoms) (37, 53). SCFAs are not only a source of energy recovered from the non-digestible diet but also important modulators of immune responses, gut barrier functions, inflammation and cardiovascular disease (36, 54). For instances, butyrate, one of the three most abundant SCFAs (acetate, propionate and butyrate), is known to control T_reg_ differentiation (55), macrophage functions (56) and promote anti-inflammatory functions of human monocytes by inducing IL-10 and inhibiting IL-12 (57). Butyrate-producing bacteria were also shown to be decreased in the gut from PLWH leading to enhanced immune activation and inflammation (58). In our studies, targeted metabolomic profiling in fecal samples collected from HIV+ and HIV^neg^ individuals showed a clear tendency for lower acetate, propionate and butyrate in individuals with subclinical atherosclerosis, however, without reaching statistical significance. Indeed, a statistical difference and subclinical CVD association was only reached with caproic (hexanoic) acid. Interestingly, caproic acid was previously shown to protect against dysbioisis and expansion of pathogenic bacteria in animals (59). In humans, fecal levels of caproic acid were recently shown to be inversely correlated with Crohn’s disease activity (60), which is a chronic inflammatory condition associated with CVD risk. Of note, we did not observe significant correlations between the decreased caproic acid levels and any of the two bacterial species showing decreased abundance in individuals with subclinical atherosclerosis; *Streptococcus sobrinus*, and *Olsenella unclassified*, although earlier studies demonstrated a link between *Olsenella* genera and caproic acid/caproate production (61). However, we acknowledge the small sample size used for the microbiome sequencing (n=16) compared to the targeted metabolome of short chain fatty acids (n=90). Further studies on large numbers of participants for the microbiome analysis are then warranted.

We further showed in the current study that conditioning of human PBMCs with this SCFA could readily diminish the LPS-mediated upregulation of the key inflammatory cytokines IL-32, IL-18 and IL-1β. The importance of decreasing these inflammatory cytokines to protect against CVD is supported by data from the CANTOS study where targeting IL-1β with the canakinumab-blocking antibody induced a decrease in atherosclerosis and adverse cardiac events (62). However, very recent data from the same study highlighted the need and importance to further block residual inflammation mediated by other cytokines such IL-18 and IL-6 (63). This suggests that residual inflammation in ART-treated individuals is multifactorial and requires novel supportive treatments having the potential to target multiple pathways at the same time. Given the larger effect of caproic acid on multiple key inflammatory cytokines including IL-32, we believe that our results pave the way for a novel, natural supplement treatment that may represent a convenient and a manageable way to counteract the residual and persistent inflammation and to protect against CVD. Yet, further studies are needed to explore the mechanisms by which caproic acid inhibits the aforementioned cytokines and whether its effect is specific for bacterial ligands (LPS) or it extends to viral and other microorganism ligands as well. In addition, further studies are also needed to investigate whether differences in the abundance of gut levels of caproic acid and/or micobiome composition might be linked with the differential expression in IL-32 between men and women that we observed as discussed above. This scenario is high likely as recent studies suggested sex-associated differences in circulating short chain fatty acids and their positive impact on decreasing blood pressure (64).

**Figure 7:**
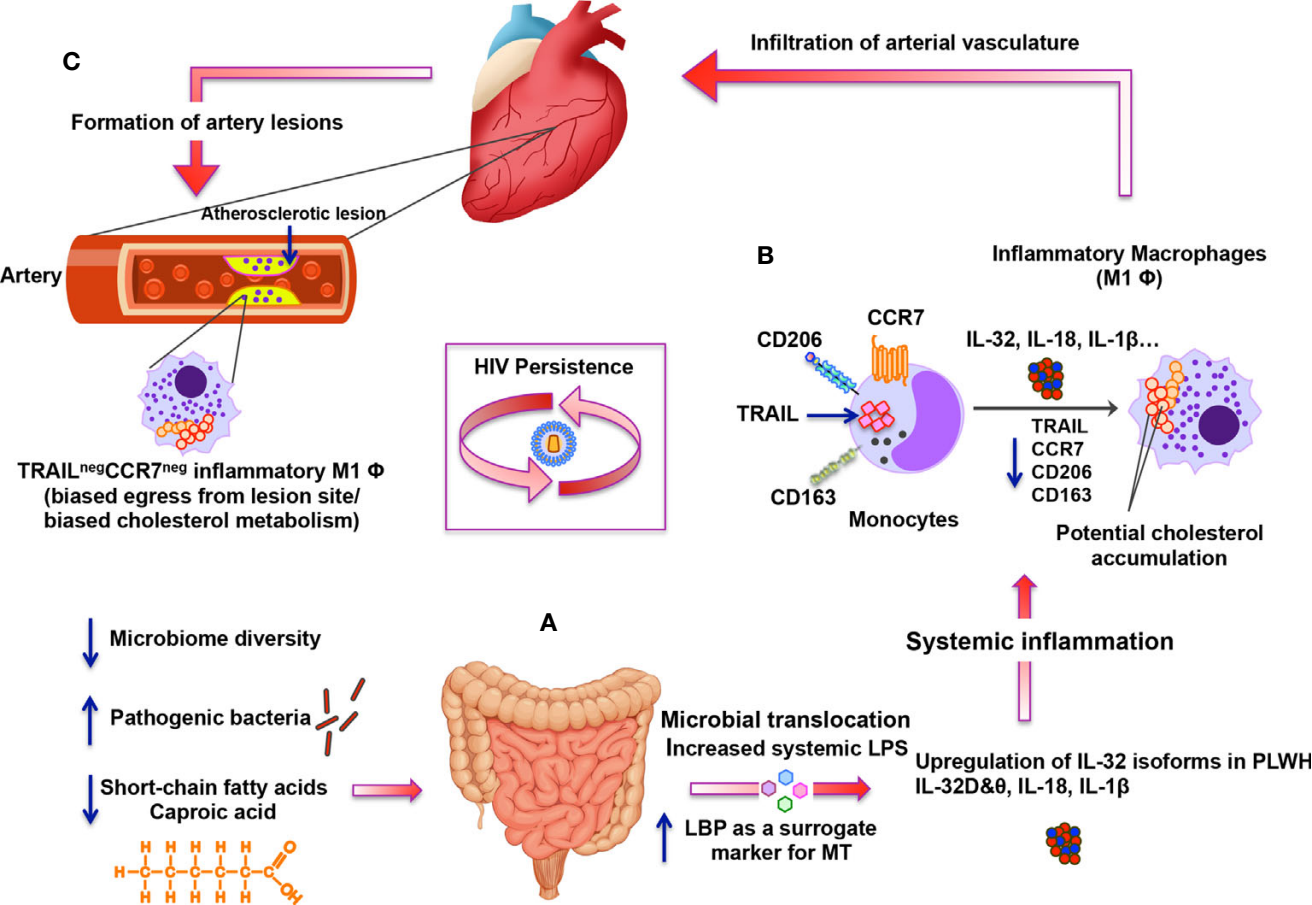
Schematic representation for the altered gut microbiome and metabolome under persistent HIV infection and their impact on systemic inflammation, IL-32 upregulation and atherosclerosis. **(A)** Under persistent HIV infection, decreased microbiome diversity and higher abundance of pathogenic bacteria combined with lower levels of short-chain fatty acids (mainly caproic acid) are associated with increased microbial translocation (MT) with high levels of systemic LPS and LBP. MT induces systemic inflammation and enhances expression of specific IL-32 isoforms together with other inflammatory cytokines such as IL-18 and IL-1β. **(B)** Specific IL-32 isoforms (IL-32β, γ and potentially IL-32D isoforms) induce the maturation of monocytes into inflammatory macrophages with M1 phenotype (CD206^neg^CD163^neg^) having decreased expression of CCR7 and TRAIL (likely mediated by up-regulation of IL-18). Inflammatory TRAIL^neg^ monocytes/macrophages are known to have biased cholesterol metabolism. **(C)** These TRAIL^neg^ monocytes/macrophages are then hypothesized to infiltrate vascular endothelium of the heart and contribute to the atherosclerotic lesion formation. IL-32-mediated down-regulation of CCR7 on TRAIL^neg^ monocytes/macrophages may also contribute to the biased egress of these cells from the lesion-forming site, which would foster atherosclerosis.

In conclusion, while the inflammatory process is an integral part of the immune response against pathogens, it is tightly regulated by self-resolving mechanisms to avoid collateral tissue-damage and cardiovascular disease. This tight regulation is compromised in HIV infection, even during ART, leading to persistent upregulation of key inflammatory cytokines such as IL-32, IL-18 and IL-1β. These cytokines impact major players of the immune response such as monocytes/macrophages by enhancing their inflammatory capacity and compromising their potential egress from the site of inflammation, thus eventually contributing to atherogenesis. As summarized in **Figure 7**, this complex process seems to be the consequence of disbalanced gut microbiota and loss of optimal gut metabolome of short-chain fatty acids that likely protect against the upregulation of inflammatory cytokines. Identification of caproic acid, a component of this metabolome, as an anti-IL-32 and anti-inflammatory player by the current study represents a key finding in the search for novel atheroprotective agents. Further studies are thus warranted to investigate the potential therapeutic potential of this metabolite.

## Data Availability Statement

The original contributions presented in the study are publicly available. This data can be found here: NCBI repository, Accession number: PRJNA713013 (https://www.ncbi.nlm.nih.gov/sra/PRJNA713013).

## Ethics Statement

The studies involving human participants were reviewed and approved by Centre Hospitalier de l’Université de Montréal, Ethical approval # CE.11.063. The patients/participants provided their written informed consent to participate in this study.

## Author Contributions

ME-F planned and performed the experiments, analyzed and interpreted the data and wrote the manuscript. MD, CC-L, MS, IB provided samples and coronary artery and carotid artery imaging data from the research subjects, contributed to the study design and discussed results. IT generated the reservoir results, contributed to study design and data analysis. EL-A, MS, SZ, RB, HR, AC, SL and JM contributed to the RNA and soluble measures. J-GB, BT, J-PR, RT, SK, RK, JK, AF and SG provided biosamples and/or and contributed to critical revision of the manuscript. EG, AF and JPG contributed to the microbiome and biomarkers analysis. PA, NC, DBH, RCK and ALL contributed to study design, data analysis and interpretation. CT is the principal investigator of the study, supervised the experiments, and contributed to data interpretation and manuscript writing. All authors contributed to the article and approved the submitted version.

## Funding

This work was supported by funds through the Canadian Institutes of Health Research, CIHR [grant number PJT 148482], National Institutes of Health, NIH [grant number R01AG054324], Fonds de Recherche Santé du Québec, FRQS [grant number 35381] and FRQS SIDA-MI network. DH is supported by NIH K award (K01-HL-137557). SL is an FRQ-S Research Scholars Emeritus awardee. NC is supported by a Research Scholar Career Award of the Quebec Health Research Fund (FRQ-S, #253292). RK is supported by 5R01MD011389, 5R01HL140976 and 1R01HL148094 from NIH.

## Conflict of Interest 

JK has stock ownership in Bristol-Myers Squibb, Johnson & Johnson, Medtronic, Merck and Pfizer. J-PG was employed by the company Caprion Inc. (now CellCarta).

The remaining authors declare that the research was conducted in the absence of any commercial or financial relationships that could be construed as a potential conflict of interest.

## References

[1] Feinstein MJ Bahiru E Achenbach C Longenecker CT Hsue P So-Armah K. Patterns of Cardiovascular Mortality for HIV-Infected Adults in the United States: 1999 to 2013. Am J Cardiol (2016) 117(2):214–20. 10.1016/j.amjcard.2015.10.030 PMC5308060 26639041

[2] Alonso A Barnes AE Guest JL Shah A Shao IY Marconi V. HIV Infection and Incidence of Cardiovascular Diseases: An Analysis of a Large Healthcare Database. J Am Heart Assoc (2019) 8(14):e012241. 10.1161/JAHA.119.012241 31266386 PMC6662120

[3] Hanna DB Ramaswamy C Kaplan RC Kizer JR Anastos K Daskalakis D. Trends in Cardiovascular Disease Mortality Among Persons With HIV in New York City, 2001-2012. Clin Infect Dis (2016) 63(8):1122–9. 10.1093/cid/ciw470 PMC5873364 27444412

[4] Lang S Mary-Krause M Cotte L Gilquin J Partisani M Simon A. French Hospital Database on H-AC. Increased risk of myocardial infarction in HIV-infected patients in France, relative to the general population. AIDS (2010) 24(8):1228–30. 10.1097/QAD.0b013e328339192f 20400883

[5] Triant VA Lee H Hadigan C Grinspoon SK. Increased acute myocardial infarction rates and cardiovascular risk factors among patients with human immunodeficiency virus disease. J Clin Endocrinol Metab (2007) 92(7):2506–12. 10.1210/jc.2006-2190 PMC2763385 17456578

[6] Durand M Sheehy O Baril JG Lelorier J Tremblay CL. Association between HIV infection, antiretroviral therapy, and risk of acute myocardial infarction: a cohort and nested case-control study using Quebec’s public health insurance database. J Acquir Immune Defic Syndr (2011) 57(3):245–53. 10.1097/QAI.0b013e31821d33a5 21499115

[7] Shah ASV Stelzle D Lee KK Beck EJ Alam S Clifford S. Global Burden of Atherosclerotic Cardiovascular Disease in People Living With HIV: Systematic Review and Meta-Analysis. Circulation (2018) 138(11):1100–12. 10.1161/CIRCULATIONAHA.117.033369 PMC6221183 29967196

[8] McGettrick P Mallon PWG Sabin CA. Cardiovascular disease in HIV patients: recent advances in predicting and managing risk. Expert Rev Anti Infect Ther (2020) 18(7):677–88. 10.1080/14787210.2020.1757430 32306781

[9] Tabas I Lichtman AH. Monocyte-Macrophages and T Cells in Atherosclerosis. Immunity (2017) 47(4):621–34. 10.1016/j.immuni.2017.09.008 PMC5747297 29045897

[10] Huang WC Sala-Newby GB Susana A Johnson JL Newby AC. Classical macrophage activation up-regulates several matrix metalloproteinases through mitogen activated protein kinases and nuclear factor-kappaB. PloS One (2012) 7(8):e42507. 10.1371/journal.pone.0042507 22880008 PMC3411745

[11] Robertson AK Hansson GK. T cells in atherogenesis: for better or for worse? Arterioscler Thromb Vasc Biol (2006) 26(11):2421–32. 10.1161/01.ATV.0000245830.29764.84 16973967

[12] Ramji DP Davies TS. Cytokines in atherosclerosis: Key players in all stages of disease and promising therapeutic targets. Cytokine Growth Factor Rev (2015) 26(6):673–85. 10.1016/j.cytogfr.2015.04.003 PMC4671520 26005197

[13] Zaidan SM Leyre L Bunet R Larouche-Anctil E Turcotte I Sylla M. Upregulation of IL-32 Isoforms in Virologically Suppressed HIV-Infected Individuals: Potential Role in Persistent Inflammation and Transcription From Stable HIV-1 Reservoirs. J Acquir Immune Defic Syndr (2019) 82(5):503–13. 10.1097/QAI.0000000000002185 PMC6857723 31714430

[14] El-Far M Kouassi P Sylla M Zhang Y Fouda A Fabre T. Investigators of the Canadian HIVSPC. Proinflammatory isoforms of IL-32 as novel and robust biomarkers for control failure in HIV-infected slow progressors. Sci Rep (2016) 6:22902. 10.1038/srep22902 26978598 PMC4792165

[15] Vaccarezza M Balla C Rizzo P. Atherosclerosis as an inflammatory disease: Doubts? No more. Int J Cardiol Heart Vasc (2018) 19:1–2. 10.1016/j.ijcha.2018.03.003 29946555 PMC6016073

[16] Damen M Popa CD Netea MG Dinarello CA Joosten LAB. Interleukin-32 in chronic inflammatory conditions is associated with a higher risk of cardiovascular diseases. Atherosclerosis (2017) 264:83–91. 10.1016/j.atherosclerosis.2017.07.005 28716457

[17] Hong JT Son DJ Lee CK Yoon DY Lee DH Park MH. Interleukin 32, inflammation and cancer. Pharmacol Ther (2017) 174:127–37. 10.1016/j.pharmthera.2017.02.025 28223235

[18] Kang JW Park YS Lee DH Kim MS Bak Y Park SH. Interleukin-32delta interacts with IL-32beta and inhibits IL-32beta-mediated IL-10 production. FEBS Lett (2013) 587(23):3776–81. 10.1016/j.febslet.2013.10.019 24396867

[19] Durand M Chartrand-Lefebvre C Baril JG Trottier S Trottier B Harris M. investigators of and aging cohort study - determinants of increased risk of cardio-vascular diseases in HIV-infected individuals: rationale and study protocol. BMC Infect Dis (2017) 17(1):611. 10.1186/s12879-017-2692-2 PMC5594495 28893184

[20] Chen Z Boldeanu I Nepveu S Durand M Chin AS Kauffmann C. In vivo coronary artery plaque assessment with computed tomography angiography: is there an impact of iterative reconstruction on plaque volume and attenuation metrics? Acta Radiol (2017) 58(6):660–9. 10.1177/0284185116664229 27650033

[21] Vandergeeten C Fromentin R Merlini E Lawani MB DaFonseca S Bakeman W. Cross-clade ultrasensitive PCR-based assays to measure HIV persistence in large-cohort studies. J Virol (2014) 88(21):12385–96. 10.1128/JVI.00609-14 PMC4248919 25122785

[22] O’Doherty U Swiggard WJ Malim MH. Human immunodeficiency virus type 1 spinoculation enhances infection through virus binding. J Virol (2000) 74(21):10074–80. 10.1128/jvi.74.21.10074-10080.2000 PMC102046 11024136

[23] Truong DT Franzosa EA Tickle TL Scholz M Weingart G Pasolli E. MetaPhlAn2 for enhanced metagenomic taxonomic profiling. Nat Methods (2015) 12(10):902–3. 10.1038/nmeth.3589 26418763

[24] Foster ZS Sharpton TJ Grunwald NJ. Metacoder: An R package for visualization and manipulation of community taxonomic diversity data. PloS Comput Biol (2017) 13(2):e1005404. 10.1371/journal.pcbi.1005404 28222096 PMC5340466

[25] Chong J Liu P Zhou G Xia J. Using MicrobiomeAnalyst for comprehensive statistical, functional, and meta-analysis of microbiome data. Nat Protoc (2020) 15(3):799–821. 10.1038/s41596-019-0264-1 31942082

[26] Han J Lin K Sequeira C Borchers CH. An isotope-labeled chemical derivatization method for the quantitation of short-chain fatty acids in human feces by liquid chromatography-tandem mass spectrometry. Anal Chim Acta (2015) 854:86–94. 10.1016/j.aca.2014.11.015 25479871

[27] Ribeiro-Dias F Saar Gomes R de Lima Silva LL Dos Santos JC Joosten LA. Interleukin 32: a novel player in the control of infectious diseases. J Leukoc Biol (2017) 101(1):39–52. 10.1189/jlb.4RU0416-175RR 27793959

[28] Tousoulis D Oikonomou E Economou EK Crea F Kaski JC. Inflammatory cytokines in atherosclerosis: current therapeutic approaches. Eur Heart J (2016) 37(22):1723–32. 10.1093/eurheartj/ehv759 26843277

[29] van der Loo M. Distribution based outlier detection for univariate data. In: Discussion paper 10003. The Hague: Statistics Netherlands (2010).

[30] Cartland SP Genner SW Martinez GJ Robertson S Kockx M Lin RC. TRAIL-Expressing Monocyte/Macrophages Are Critical for Reducing Inflammation and Atherosclerosis. iScience (2019) 12:41–52. 10.1016/j.isci.2018.12.037 30665196 PMC6348195

[31] Brenchley JM Paiardini M Knox KS Asher AI Cervasi B Asher TE. Differential Th17 CD4 T-cell depletion in pathogenic and nonpathogenic lentiviral infections. Blood (2008) 112(7):2826–35. 10.1182/blood-2008-05-159301 PMC2556618 18664624

[32] El-Far M Tremblay CL. Gut microbial diversity in HIV infection post combined antiretroviral therapy: a key target for prevention of cardiovascular disease. Curr Opin HIV AIDS (2018) 13(1):38–44. 10.1097/COH.0000000000000426 29045253 PMC5718258

[33] Netea MG Azam T Lewis EC Joosten LA Wang M Langenberg D. Mycobacterium tuberculosis induces interleukin-32 production through a caspase- 1/IL-18/interferon-gamma-dependent mechanism. PloS Med (2006) 3(8):e277. 10.1371/journal.pmed.0030277 16903774 PMC1539091

[34] Tuddenham SA Koay WLA Zhao N White JR Ghanem KG Sears CL. Consortium HIVMR-a. The Impact of Human Immunodeficiency Virus Infection on Gut Microbiota alpha-Diversity: An Individual-level Meta-analysis. Clin Infect Dis (2020) 70(4):615–27. 10.1093/cid/ciz258 PMC7319268 30921452

[35] Kasubuchi M Hasegawa S Hiramatsu T Ichimura A Kimura I. Dietary gut microbial metabolites, short-chain fatty acids, and host metabolic regulation. Nutrients (2015) 7(4):2839–49. 10.3390/nu7042839 PMC4425176 25875123

[36] Chambers ES Preston T Frost G Morrison DJ. Role of Gut Microbiota-Generated Short-Chain Fatty Acids in Metabolic and Cardiovascular Health. Curr Nutr Rep (2018) 7(4):198–206. 10.1007/s13668-018-0248-8 30264354 PMC6244749

[37] Ohira H Tsutsui W Fujioka Y. Are Short Chain Fatty Acids in Gut Microbiota Defensive Players for Inflammation and Atherosclerosis? J Atheroscler Thromb (2017) 24(7):660–72. 10.5551/jat.RV17006 PMC5517538 28552897

[38] Li W Liu Y Mukhtar MM Gong R Pan Y Rasool ST. Activation of interleukin-32 pro-inflammatory pathway in response to influenza A virus infection. PloS One (2008) 3(4):e1985. 10.1371/journal.pone.0001985 18414668 PMC2288676

[39] Li W Yang F Liu Y Gong R Liu L Feng Y. Negative feedback regulation of IL-32 production by iNOS activation in response to dsRNA or influenza virus infection. Eur J Immunol (2009) 39(4):1019–24. 10.1002/eji.200838885 19291698

[40] Choi JD Bae SY Hong JW Azam T Dinarello CA Her E. Identification of the most active interleukin-32 isoform. Immunology (2009) 126(4):535–42. 10.1111/j.1365-2567.2008.02917.x PMC2673365 18771438

[41] Son DJ Jung YY Seo YS Park H Lee DH Kim S. Interleukin-32alpha Inhibits Endothelial Inflammation, Vascular Smooth Muscle Cell Activation, and Atherosclerosis by Upregulating Timp3 and Reck through suppressing microRNA-205 Biogenesis. Theranostics (2017) 7(8):2186–203. 10.7150/thno.18407 PMC5505053 28740544

[42] Kim MS Kang JW Lee DH Bak Y Park YS Song YS. IL-32theta negatively regulates IL-1beta production through its interaction with PKCdelta and the inhibition of PU.1 phosphorylation. FEBS Lett (2014) 588(17):2822–9. 10.1016/j.febslet.2014.06.029 24996056

[43] Kim SH Han SY Azam T Yoon DY Dinarello CA. Interleukin-32: a cytokine and inducer of TNFalpha. Immunity (2005) 22(1):131–42. 10.1016/j.immuni.2004.12.003 15664165

[44] Mallat Z Corbaz A Scoazec A Besnard S Leseche G Chvatchko Y. Expression of interleukin-18 in human atherosclerotic plaques and relation to plaque instability. Circulation (2001) 104(14):1598–603. 10.1161/hc3901.096721 11581135

[45] Libby P. Interleukin-1 Beta as a Target for Atherosclerosis Therapy: Biological Basis of CANTOS and Beyond. J Am Coll Cardiol (2017) 70(18):2278–89. 10.1016/j.jacc.2017.09.028 PMC5687846 29073957

[46] Christgen S Place DE Kanneganti TD. Toward targeting inflammasomes: insights into their regulation and activation. Cell Res (2020) 30(4):315–27. 10.1038/s41422-020-0295-8 PMC7118104 32152420

[47] Zhou W Chen C Chen Z Liu L Jiang J Wu Z. NLRP3: A Novel Mediator in Cardiovascular Disease. J Immunol Res (2018) 2018:5702103. 10.1155/2018/5702103 29850631 PMC5911339

[48] Trogan E Feig JE Dogan S Rothblat GH Angeli V Tacke F. Gene expression changes in foam cells and the role of chemokine receptor CCR7 during atherosclerosis regression in ApoE-deficient mice. Proc Natl Acad Sci U.S.A. (2006) 103(10):3781–6. 10.1073/pnas.0511043103 PMC1450154 16537455

[49] Feig JE Shang Y Rotllan N Vengrenyuk Y Wu C Shamir R. Statins promote the regression of atherosclerosis via activation of the CCR7-dependent emigration pathway in macrophages. PloS One (2011) 6(12):e28534. 10.1371/journal.pone.0028534 22163030 PMC3232231

[50] Nowak P Troseid M Avershina E Barqasho B Neogi U Holm K. Gut microbiota diversity predicts immune status in HIV-1 infection. AIDS (2015) 29(18):2409–18. 10.1097/QAD.0000000000000869 26355675

[51] Thota VR Dacha S Natarajan A Nerad J. Eggerthella lenta bacteremia in a Crohn’s disease patient after ileocecal resection. Future Microbiol (2011) 6(5):595–7. 10.2217/fmb.11.31 21585265

[52] Salem F Kindt N Marchesi JR Netter P Lopez A Kokten T. Gut microbiome in chronic rheumatic and inflammatory bowel diseases: Similarities and differences. United Eur Gastroenterol J (2019) 7(8):1008–32. 10.1177/2050640619867555 PMC6794689 31662859

[53] Cook SI Sellin JH. Review article: short chain fatty acids in health and disease. Aliment Pharmacol Ther (1998) 12(6):499–507. 10.1046/j.1365-2036.1998.00337.x 9678808

[54] Sun M Wu W Liu Z Cong Y. Microbiota metabolite short chain fatty acids, GPCR, and inflammatory bowel diseases. J Gastroenterol (2017) 52(1):1–8. 10.1007/s00535-016-1242-9 27448578 PMC5215992

[55] Furusawa Y Obata Y Fukuda S Endo TA Nakato G Takahashi D. Commensal microbe-derived butyrate induces the differentiation of colonic regulatory T cells. Nature (2013) 504(7480):446–50. 10.1038/nature12721 24226770

[56] Chang PV Hao L Offermanns S Medzhitov R. The microbial metabolite butyrate regulates intestinal macrophage function via histone deacetylase inhibition. Proc Natl Acad Sci U.S.A. (2014) 111(6):2247–52. 10.1073/pnas.1322269111 PMC3926023 24390544

[57] Saemann MD Bohmig GA Osterreicher CH Burtscher H Parolini O Diakos C. Anti-inflammatory effects of sodium butyrate on human monocytes: potent inhibition of IL-12 and up-regulation of IL-10 production. FASEB J (2000) 14(15):2380–2. 10.1096/fj.00-0359fje 11024006

[58] Dillon SM Kibbie J Lee EJ Guo K Santiago ML Austin GL. Low abundance of colonic butyrate-producing bacteria in HIV infection is associated with microbial translocation and immune activation. AIDS (2017) 31(4):511–21. 10.1097/QAD.0000000000001366 PMC5263163 28002063

[59] Van Immerseel F De Buck J Boyen F Bohez L Pasmans F Volf J. Medium-chain fatty acids decrease colonization and invasion through hilA suppression shortly after infection of chickens with Salmonella enterica serovar Enteritidis. Appl Environ Microbiol (2004) 70(6):3582–7. 10.1128/AEM.70.6.3582-3587.2004 PMC427757 15184160

[60] De Preter V Machiels K Joossens M Arijs I Matthys C Vermeire S. Faecal metabolite profiling identifies medium-chain fatty acids as discriminating compounds in IBD. Gut (2015) 64(3):447–58. 10.1136/gutjnl-2013-306423 24811995

[61] Lambrecht J Cichocki N Schattenberg F Kleinsteuber S Harms H Muller S. Key sub-community dynamics of medium-chain carboxylate production. Microb Cell Fact (2019) 18(1):92. 10.1186/s12934-019-1143-8 31138218 PMC6537167

[62] Ridker PM Everett BM Thuren T MacFadyen JG Chang WH Ballantyne C. Antiinflammatory Therapy with Canakinumab for Atherosclerotic Disease. N Engl J Med (2017) 377(12):1119–31. 10.1056/NEJMoa1707914 28845751

[63] Ridker PM MacFadyen JG Thuren T Libby P. Group obotCT. Residual inflammatory risk associated with interleukin-18 and interleukin-6 after successful interleukin-1beta inhibition with canakinumab: further rationale for the development of targeted anti-cytokine therapies for the treatment of atherothrombosis. Eur Heart J (2019) 41(23):2153–63. 10.1093/eurheartj/ehz542 31504417

[64] Chen L He FJ Dong Y Huang Y Wang C Harshfield GA. Modest Sodium Reduction Increases Circulating Short-Chain Fatty Acids in Untreated Hypertensives: A Randomized, Double-Blind, Placebo-Controlled Trial. Hypertension (2020) 76(1):73–9. 10.1161/HYPERTENSIONAHA.120.14800 PMC7328301 32475312

