# Supplementary material for: Upregulated IL-32 Expression And Reduced Gut Short Chain Fatty Acid Caproic Acid in People Living With HIV With Subclinical Atherosclerosis
Source: Front Immunol. 2021 Apr 15;12:664371. doi: 10.3389/fimmu.2021.664371 (PMC8083984; doi:10.3389/fimmu.2021.664371)
Supplement: Supplementary file 1 [file DataSheet_1.pdf]

Supplementary Material

Supplemental Table 1: Panel of plasma analytes from HIV+ and HIV^{neg} with and without subclinical atherosclerosis.

Analytes (% of detectable samples: HIV+/HIV^{neg})

**IL-8**	**IL-18**	**IL-1RA**	**Ghrelin (total)**	**MIP-1α**	**LBP**	**Ghrelin (Active)**
(100%/100%)	(100%/100%)	(100%/100%)	(100%/100%)	(96%/100%)	(77%/33%)	(24%/22%)
**GIP (total)**	**IL-7**	**IL-2Ra**	**Glucagon**	**IFN-γ**	**I-FABP**	**IL-2**
(100%/100%)	(100%/100%)	(100%/100%)	(100%/100%)	(95%/96%)	(77%/33%)	(18%/37%)
**Insulin**	**TPO**	**IL-9**	**LH**	**GIP (active)**	**IL-29**	**IL-31**
(100%/100%)	(100%/100%)	(100%/100%)	(100%/100%)	(94%/94%)	(73%/33%)	(16%/6%)
**VEGF-A**	**CTACK**	**b-NGF**	**PP**	**IL-6**	**TNF-β**	**Active GLP-1**
(100%/100%)	(100%/100%)	(100%/100%)	(100%/100%)	(92%/94%)	(72%/63%)	(13%/20%)
**Eotaxin**	**ENA-78**	**BAFF**	**PYY (total)**	**GIP (inactive)**	**IL-17A**	**IL-17A/F**
(100%/100%)	(100%/100%)	(100%/100%)	(100%/100%)	(92%/96%)	(68%/67%)	(8%/16%)
**IP-10**	**Fractalkine**	**Eotaxin-2**	**LL-37**	**TSLP**	IL-23	**IL-33**
(100%/100%)	(100%/100%)	(100%/100%)	(100%/98%)	(89%/88%)	(68%/63%)	(8%/8%)
**MCP-1**	**IL-22**	**FGF-23**	**C-Peptide**	**GLP-1 (inactive)**	**IL-17E**	**IFN-α2a**
(100%/100%)	(100%/100%)	(100%/100%)	(99%/100%)	[illegible]	(66%/88%)	(6%/6%)
**MCP-4**	**IL-27**	**CXCL-1**	**TNF-α**	**BDNF**	**IL-13**	**IL-12p70**
(100%/100%)	(100%/100%)	(100%/100%)	(99%/100%)	(84%/57%)	(65%/69%)	(5%/22%)
**MDC**	**CXCL12**	**M-CSF**	**Proinsulin**	G-CSF	**IL-17D**	**IL-4**
(100%/100%)	(100%/100%)	(100%/100%)	(99%/100%)	**(82%/76%)**	(58%/27%)	(1%/4%)
**TARC**	**GLP-1 (total)**	**MCP-2**	IL-1α	**IL-21**	**IFN-β**	**GM-CSF**
(100%/100%)	(100%/100%)	(100%/100%)	**(99%/96%)**	(82%/73%)	(56%/92%)	(0%/1%)
**IL-12/IL-23p40**	**EPO**	**TRAIL**	**I-309**	**IL-17F**	**IL-10**	**IL-3**
(100%/100%)	(100%/100%)	(100%/100%)	(99%/100%)	(81%/65%)	(48%/20%)	(44%/82%)
**IL-15**	**FLT3L**	**FGF-21**	**Leptin**	**CCL26**	**IL-5**	**sCD14**
(100%/100%)	(100%/100%)	(100%/100%)	(99%/100%)	(80%/84%)	(47%/27%)	(78%/35%)
**IL-16**	**IL-17C**	**FSH**	**IL-1β**			
(100%/100%)	(100%/100%)	(100%/100%)	(96%/90%)			

Supplemental Table 2. Sequences of primer sets and probes used for nested quantitative PCR of integrated and total HIV DNA, HIV Gag RNA and the surrogate human CD3 gene.

Target	Step	Name of primer probe	Sequence (5' to 3')
**Total HIV DNA and LTR Gag RNA**	**Pre amplification**	ULF1	ATG CCA CGT AAG CGA AAC TCT GGG TCT CTC TDG TTA GAC
		UR1	CCA TCT CTC TCC TTC TAG C
	**Quantitative PCR**	LambdaT UR2	ATG CCA CGT AAG CGA AAC T CTG AGG GAT CTC TAG TTA CC
		UHIV Taqman	/56-FAM/CA CTC AAG G/ZEN/C AAG CTT TAT TGA GGC /3IABkFQ/
**Integrated HIV DNA**	**Pre amplification**	ULF1	ATG CCA CGT AAG CGA AAC TCT GGG TCT CTC TDG TTA GAC
		Alu1	TCC CAG CTA CTG GGG AGG CTG AGG
		Alu2	GCC TCC CAA AGT GCT GGG ATT ACA G
	**Quantitative PCR**	LambdaT UR2	ATG CCA CGT AAG CGA AAC T CTG AGG GAT CTC TAG TTA CC
		UHIV Taqman	/56-FAM/CA CTC AAG G/ZEN/C AAG CTT TAT TGA GGC /3IABkFQ/
**CD3**	**Pre amplification**	HCD3OUT5'	ACT GAC ATG GAA CAG GGG AAG
		HCD3OUT3'	CCA GCT CTG AAG TAG GGA ACA TAT
	**Quantitative PCR**	HCD3IN5'	GGC TAT CAT TCT TCT TCA AGG T
		HCD3IN3'	CCT CTC TTC AGC CAT TTA AGT A
		CD3 Taqman	/56-FAM/AG CAG AGA A/ZEN/C AGT TAA GAG CCT CCA T/3IABkFQ/

Supplemental Table 3: Significantly enriched or decreased microbial species in HIV+ relative to HIV^{neg} individuals (i) or in HIV+CVD+ relative to $HIV+CVD^{neg}$ (ii). Non-parametric Mann-Whitney analysis was used to generate the *p* values.

**Species**<br>**i) HIV+ *versus* HIV^{neg}**	**Phylum**	**Enriched /decreased**	***P* value**
Methanosphaera_stadtmanae	*Euryarchaeota*	Decreased	0,0005
Peptostreptococcaceae_noname	*Firmicutes*	Decreased	0,0005
Ruminococcus_callidus	*Firmicutes*	Decreased	0,0038
Olsenella_unclassified	*Actinobacteria*	Decreased	0,0038
Eubacterium_siraeum	*Firmicutes*	Decreased	0,0148
Leuconostoc_gelidum	*Firmicutes*	Decreased	0,0500
Fusobacterium_gonidiaformans	*Fusobacteria*	Decreased	0,0153
Butyrivibrio_crossotus	*Firmicutes*	Decreased	0,0203
Mitsuokella_unclassified	*Firmicutes*	Decreased	0,0132
Odoribacter_unclassified	*Bacteroidetes*	Decreased	0,0269
Mitsuokella_multacida	*Firmicutes*	Decreased	0,0170
Bifidobacterium_adolescentis	*Actinobacteria*	Decreased	0,0203
Coprococcus_comes	*Firmicutes*	Decreased	0,0582 (NS)
Ruminococcus_gnavus	*Firmicutes*	Enriched	0,0599 (NS)
Flavonifractor_plautii	*Firmicutes*	Enriched	0,0049
Streptococcus_vestibularis	*Firmicutes*	Enriched	0,0335
Eggerthella_lenta	*Actinobacteria*	Enriched	0,0599
**ii) HIV+CVD+ *versus* $HIV+CVD^{neg}$**			
Olsenella_unclassified	*Actinobacteria*	Decreased	0,0215
Streptococcus_sobrinus	*Firmicutes*	Decreased	0,0455
Phascolarctobacterium_succinatutens	*Firmicutes*	Decreased	0,0543 (NS)
Rothia_mucilaginosa	*Actinobacteria*	Enriched	0,0455
Eggerthella_unclassified	*Actinobacteria*	Enriched	0,0455

Supplemental Table 4: Demographic and clinical parameters of study participants in the faecal collection. Data collected at study entry. Numbers are shown in Mean ± SD. N/A: Non-applicable. NA: Non-available.

Variable	HIV^{neg} CVD- (Female/Male) (n=5/ n=11)	HIV^{neg}CVD+ (Female/Male) (n=6/ n=13)	*P* value	HIV+CVD- (Female/Male) (n=2/ n=15)	HIV+ CVD+ (Female/Male) (n=3/ n=35)	*P* value
Age (Years)	54.49±6.75	62,4±8.38	0.006	51.97±5.54	54.83±6.63	NS
Predicted 10 years Framingham Risk score	8.75±4.28	12±4.86	0.048	8.88±3.33	10.13±5.92	NS
Intravenous drug injection						
Injection	0 (0%)	0 (0%)		0 (0%)	7 (18.4%)	
No-injection	17 (100%)	19 (100%)		17 (100%)	31 (81.6%)	
Duration of ART (Years)	N/A	N/A		12.3±7.58	14.08±13.39	NS
Viral load (Copies/ml)	N/A	N/A		< 40	< 40	
Nadir CD4 count ($Cells/mm^3$)	N/A	N/A		152.05±111.14	203.78±139.09	NS
CD4 count ($cells/mm^3$)	NA	NA		609±277	597±256	NS
CD4/CD8 ratio	NA	NA		0.84±0.47	0.85±0.42	NS
D-dimer (mg/L)	0.271±0.160	0.371±0.112	NS	0.416±0.349	0.340±0.166	NS
hsCRP (mg/L)	NA	NA		6.12±5.17	4.64±1.77	NS
LDL–C** (mmol/L)	3.31±0.88	3.13±0.64	NS	2.89±0.76	2.71±0.93	NS
HDL–C** (mmol/L)	1.49±0.49	1.5±0.27	NS	1.13±0.19	1.28±0.33	NS

El-Far et al., Supplemental Figure 1

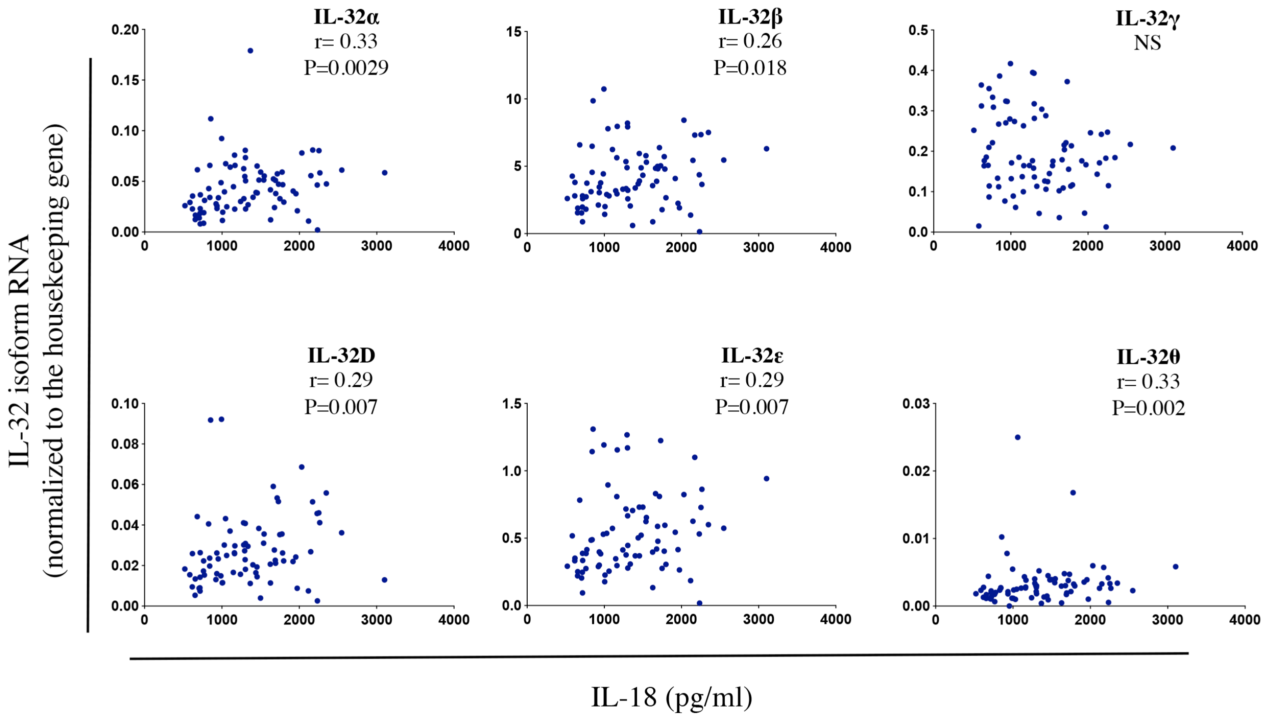

Supplemental Figure 1: Correlations between plasma IL-18 and IL-32 isoforms expression. IL-18 was measured with Meso Scale technology from plasma of HIV+ men with or without subclinical atherosclerosis (n=79). IL-32 mRNA of the different isoforms was quantified in total PBMCs from the same individuals at the same clinical visit and normalized to the house keeping gene β-glucuronidase. Data analysed with the non-parametric Spearman test.

El-Far et al., Supplemental Figure 2

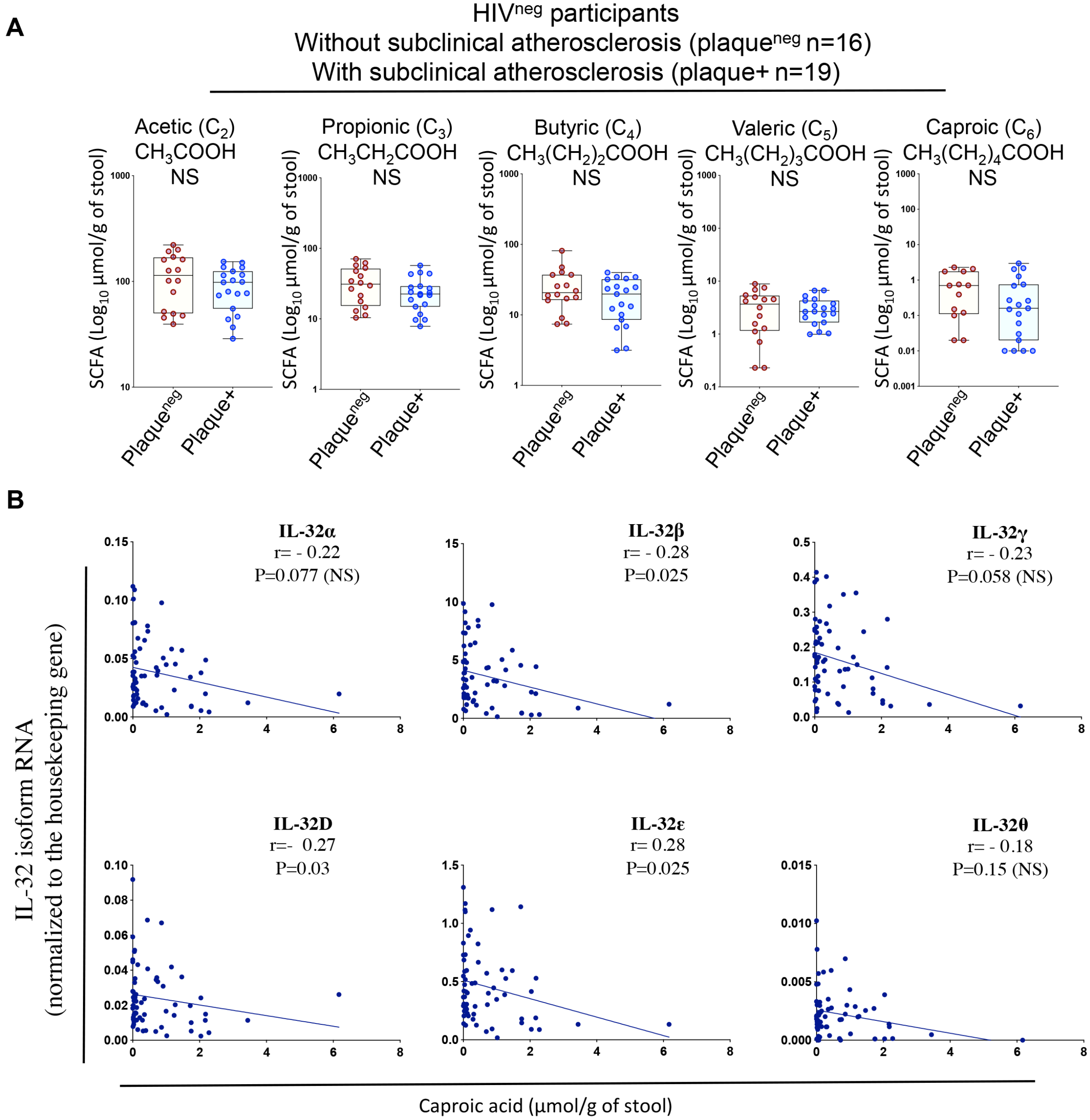

Supplemental Figure 2: Gut levels of the Short-chain fatty acids from HIV^{neg} individuals with or without subclinical CVD and correlations with IL-32 RNA. A) Levels of the individual short-chain fatty acids measured in faecal samples collected from HIV^{neg}Plaque^{neg} (n=16) compared to HIV^{neg}Plaque+ (n=19). B) Correlation between levels of caproic acid in faecal samples and individual IL-32 isoforms mRNA (measured by RT-qPCR and normalized to the housekeeping gene β-glucuronidase) from HIV+ and HIV^{neg} individuals (n=63). Data analysed with the non-parametric Mann-Whitney test in A and non-parametric Spearman test in B. *M: Median, *Q1: first quartile, *Q3: third quartile.
